# Contribution of endometrial microbiome to inflammation-mediated infertility in women undergoing ART

**DOI:** 10.1093/humrep/deaf252

**Published:** 2026-02-03

**Authors:** F Giangrazi, J A Sugrue, V M Sularea, A A I Brugman, M Horan, M Wingfield, D A Crosby, L E Glover, C O’Farrelly

**Affiliations:** School of Biochemistry and Immunology, Trinity Biomedical Sciences Institute, Trinity College Dublin, Dublin, Ireland; School of Biochemistry and Immunology, Trinity Biomedical Sciences Institute, Trinity College Dublin, Dublin, Ireland; Translational Immunology Unit, Institut Pasteur, Paris, France; School of Biochemistry and Immunology, Trinity Biomedical Sciences Institute, Trinity College Dublin, Dublin, Ireland; School of Biochemistry and Immunology, Trinity Biomedical Sciences Institute, Trinity College Dublin, Dublin, Ireland; Department of Obstetrics and Gynaecology, National Maternity Hospital, Dublin, Ireland; Department of Reproductive Medicine, Merrion Fertility Clinic, Dublin, Ireland; Department of Reproductive Medicine, Merrion Fertility Clinic, Dublin, Ireland; School of Medicine, University College Dublin, Dublin, Ireland; Department of Obstetrics and Gynaecology, National Maternity Hospital, Dublin, Ireland; Department of Reproductive Medicine, Merrion Fertility Clinic, Dublin, Ireland; School of Medicine, University College Dublin, Dublin, Ireland; Department of Reproductive Medicine, Merrion Fertility Clinic, Dublin, Ireland; School of Medicine, University College Dublin, Dublin, Ireland; School of Medicine, Trinity College Dublin, Dublin, Ireland; School of Biochemistry and Immunology, Trinity Biomedical Sciences Institute, Trinity College Dublin, Dublin, Ireland; School of Medicine, Trinity College Dublin, Dublin, Ireland

**Keywords:** endometrial receptivity, microbiome, endometrium, endometrial epithelial cells, female infertility, assisted reproduction, 16S-sequencing, inflammation, unexplained infertility

## Abstract

**STUDY QUESTION:**

Is the endometrial microbiome altered in women who fail to get pregnant after ART and do microbial-derived metabolites influence endometrial cellular mechanisms important for embryo implantation?

**SUMMARY ANSWER:**

The endometrial microbiome in women who fail to get pregnant after ART is more diverse and has fewer lactobacilli species than the endometrial microbiomes of women who become pregnant; the short-chain fatty acid butyrate, a common metabolite found in the presence of increased microbial diversity, diminishes endometrial epithelial barrier function and increases the expression of inflammatory markers.

**WHAT IS KNOWN ALREADY:**

Shifts in the endometrial microbial community structure have been linked to fertility and pregnancy complications although the underlying mechanisms are poorly understood. Microbial metabolites at other mucosal surfaces, such as the gut, act as important modulators of immune and barrier function, particularly in epithelial cells. Effects of changes in local bacterial microbial populations on fertility, and how their metabolites might influence endometrial cell function have not been explored.

**STUDY DESIGN, SIZE, DURATION:**

In this prospective longitudinal study of ART outcomes, 29 nulliparous women with unexplained infertility were recruited between October 2016 and February 2018. Endometrial tissue samples were taken for microbiome analysis and endometrial transcriptomics prior to ART. For primary cell culture studies, endometrial biopsies were obtained from fertile women of reproductive age undergoing laparoscopic surgical investigation between February 2021 and September 2023. *In vitro* models of implantation were established using endometrial cell lines and primary endometrial stromal cells.

**PARTCIPANTS/MATERIALS, SETTING, METHODS:**

Microbiome 16S sequencing analysis was performed on bacterial DNA isolated from endometrial biopsies and correlated with receptivity markers. Endometrial RNA sequencing data from women undergoing ART were used to analyse differential gene expression of receptivity and decidualization markers in women who had a positive or negative ART cycle outcome. *In vitro* models, using both established endometrial cell lines and primary human endometrial epithelial cells and stromal cells, were developed to investigate the effects of microbial-derived metabolites. An *in vitro* model of peri-implantation was used to test the effect of butyrate on endometrial epithelial receptivity and stromal cell decidualization.

**MAIN RESULTS AND THE ROLE OF CHANCE:**

Endometrial microbiome 16S sequencing revealed a lower abundance of *Lactobacillus* spp. and significantly higher abundance of pathogenic species such as *Prevotella* spp. and *Corynebacterium* spp. in women who did not become pregnant after ART. Endometrial microbiota from women who had positive ART outcomes showed significantly lower diversity indices. Intriguingly, analysis of endometrial RNA sequencing data from women with unexplained infertility undergoing ART showed that negative ART outcomes were associated with higher levels of some receptivity and decidualization markers in their endometrial tissue. Butyrate, but not lactate or acetate, also increased some markers of epithelial receptivity and stromal decidualization. Butyrate exposure also activated defence mechanisms in cultured endometrial epithelial cells by inducing expression of antimicrobial peptide(s) and inflammation markers, as well as impairing the barrier integrity of endometrial epithelial cell monolayers.

**LARGE SCALE DATA:**

The RNA-seq data used for the study can be found in GEO database, GEO ID GSE144895. The data for the 16S sequencing can be accessed in SRA BioProject number PRJNA1338067.

**LIMITATIONS, REASONS FOR CAUTION:**

Limitations of our study include the cohort size and technical challenges that precluded absolute butyrate measurement in endometrial tissue biopsies. Biopsy collection from women undergoing gynaecological investigation varied in menstrual cycle staging and fertility diagnoses, which may contribute to the variability between responses obtained from *in vitro* stimulations. The transferred embryos were not genetically tested, but were all of good or top quality.

**WIDER IMPLICATIONS OF THE FINDINGS:**

Our findings indicate that the endometrial microbiome is altered in women who fail to become pregnant after ART, and that the microbial-derived metabolite butyrate can induce inflammation and impair endometrial epithelial barrier function and drive increased gene expression levels of markers for epithelial receptivity and stromal decidualization in *in vitro* models of peri-implantation. Endometrial microbial dysbiosis and higher expression of receptivity markers were found in women who failed to establish pregnancy post-ART. Negative ART outcomes in this cohort were found to correlate with the presence of a wider, more diverse microbial community that includes *Prevotella* spp., which is among the butyrate-producing bacteria. Further investigation of the microbial metabolome in healthy endometrium would help clarify the physiological role of butyrate and other bacterial metabolites in endometrial function.

**STUDY FUNDING/COMPETING INTEREST(S):**

This research was supported by the Grant for Fertility Innovation from Merck KGaA, grant award number 15692. The authors declare that they have no known competing financial interests or personal relationships that could have appeared to influence the work reported in this paper.

**TRIAL REGISTRATION NUMBER:**

N/A.

## Introduction

Infertility, defined in heterosexual couples as inability to conceive within 12 months of unprotected intercourse, is a major burden which can affect up to 15% of couples (ACOG Committee, 2019). While male- or female-derived factors contributing to infertility can be identified in 70–80% of infertility cases, in 20–30% of cases, couples fail to conceive while presenting with normal ovarian function, normal genito-urinary anatomy, normal testicular function and a normal ejaculate. This is designated unexplained infertility and is extremely distressing for couples and frustrating for reproductive specialists (Zegers-Hochschild *et al.*, 2017; ACOG Committee, 2019). The development of ART has improved the chances of conceiving in infertile couples, however, failure in embryo implantation still poses major limitations (Wang *et al.*, 2020). Correct embryo implantation requires a complex crosstalk between the invading embryo and the receptive endometrium. It is therefore essential to synchronize embryo transfer with the opening of the endometrial window of implantation (WOI) (Valdes *et al.*, 2017; Sebastian-Leon *et al.*, 2018). To help identify the opening of the WOI, transcriptomic approaches such as the endometrial receptivity assay (ERA) have been used to assess the expression of genes known to be induced by progesterone when the endometrium becomes receptive (Díaz-Gimeno *et al.*, 2011).

Inflammatory signalling in the endometrium plays a critical role in successful embryo implantation. An influx of uterine immune cells and remodelling of the endometrial vascular bed coincide with invasion of embryo-derived trophoblast cells into the decidua post-implantation (Gellersen *et al.*, 2007). Depletion of key pro-inflammatory pathways has been shown to negatively impact early pregnancy establishment (Hanna *et al.*, 2006; Schumacher *et al.*, 2018). Dysregulated endometrial inflammation via alterations in the pro-inflammatory IL-17 pathway, has been linked to subfertility (Ahn *et al.*, 2015; Xu *et al.*, 2016; Borkner *et al.*, 2021). We have also demonstrated dysregulation of the IL-17A pathway in endometrial tissue, together with increased circulating IL-17A protein in women with unexplained infertility undergoing ART who had an unsuccessful outcome (Crosby *et al.*, 2020). A key function of IL-17 is to coordinate host defence responses against fungal and bacterial infection at mucosal barrier sites, by driving expression of innate antimicrobial immune mediators, as well as promoting cellular proliferation, repair and development (McGeachy *et al.*, 2019). The mechanisms by which endometrial IL-17 pathway activation might be involved in implantation and pregnancy survival have not been extensively explored.

The contribution of the gut microbiome to normal physiological function of the gastrointestinal tract is well described and microbiome abnormalities are known to be key to the pathogenesis of inflammatory bowel disease and other Gastrointestinal (GI) disorders. However, the role that the uterine microbiome plays in implantation and pregnancy success is less explored. Bacterial biomass in the uterine cavity is reportedly around 10^2^–10^4^ times less than that in the vagina (Mitchell *et al.*, 2015; Chen *et al.*, 2017), and defining the normal or eubiotic endometrial microbiome has proven challenging, as evident from the marked heterogeneity between profiling studies of resident phyla (Baker *et al.*, 2018). Nevertheless, reports describe an endometrial microbiome dominated solely by *Lactobacillus* spp. (Mitchell *et al.*, 2015; Khan *et al.*, 2016; Moreno *et al.*, 2016), while others have reported a more diverse microbial composition with a higher abundance of non-lactobacilli genus (Franasiak *et al.*, 2016; Chen *et al.*, 2017; Sola-Leyva *et al.*, 2021), including *Flavobacterium* (Franasiak *et al.*, 2016), *Pseudomonas*, *Acinetobacter*, *Vagococcus* and *Sphingobium* (Chen *et al.*, 2017). Ovarian stimulation and progesterone supplementation in assisted reproduction cycles were associated with decreased *Lactobacilli* spp. and an increase in *Prevotella* and *Aptobium* spp. abundance, as well as increased bacterial biodiversity (Carosso *et al.*, 2020).

In keeping with a lack of consensus on what constitutes a healthy endometrial microbiome, the composition of the microbial metabolome is also largely unknown. In the vagina, lactic acid is the most abundant (∼110 mM) metabolite produced by a healthy microbiome (Amabebe and Anumba, 2018), and the resultant acidic pH of 3.5–3.9 promotes homeostasis and eubiosis (Aldunate *et al.*, 2015). Conversely, the local pH in the endometrium is higher and varies throughout the menstrual cycle (Feo, 1955; Ng *et al.*, 2018), suggesting a lower lactic acid content compared to vaginal tissue. Elevated levels of lactic acid or lactate in the endometrium (>1 ppm) correlate with endometriosis and unsuccessful embryo transfer in women with recurrent implantation failure (Ersahin *et al.*, 2017; Yurci *et al.*, 2021).

Metabolomic studies of healthy vaginal fluid have revealed low levels of short-chain fatty acids (SCFA) such as acetate, propionate, butyrate and succinate (Preti and Huggins, 1975; Spiegel *et al.*, 1980). SCFA are produced by the fermentation of polysaccharides such as glucose, galactose and fucose by anaerobic bacteria (Amabebe and Anumba, 2020). In bacterial vaginosis (BV), a decrease in lactic acid-producing microbiota and elevated pH > 4.5 promotes a shift towards SCFA production by increased anaerobic bacteria such as Gardnerella, Atopobium and Prevotella (Spiegel *et al.*, 1980; Aldunate *et al.*, 2015). Studies of BV-associated microbial signatures reveal acetate as the predominant metabolite (120 mM), followed by lactate (<20 mM)(Al-Mushrif *et al.*, 2000), and propionate and butyrate, albeit at much lower concentrations of <2–4 mM (Mirmonsef *et al.*, 2011).

SCFA have been perhaps best characterized in the gut, where they act both as a primary energy source for epithelia and as important immunomodulatory factors at the mucosal barrier. SCFA can be actively transported into intestinal epithelia and immune cells via monocarboxylate transporters MCT1 and MCT4. Molecular targets of SCFA include the G-protein coupled receptors GPR41 and GPR43, and receptor binding induces AMP- and phospholipase C-dependent pathways (Parada Venegas *et al.*, 2019). Butyrate promotes gut barrier function by inducing tight junction protein expression, leading to increased transepithelial electrical resistance (TEER) (Valenzano *et al.*, 2015; Zheng *et al.*, 2017), and drives an anti-inflammatory signature in both intestinal epithelial and immune cells (Thangaraju *et al.*, 2009). In the vaginal tract, SCFAs contribute to sustained dysbiosis by increasing the pH and by favouring polymicrobial growth through a mutualistic metabolite exchange that favours pathogenic species takeover (Pybus and Onderdonk, 1997). Vaginal epithelial cell stimulation with BV-associated bacterial strains also induces pro-inflammatory cytokine release (IL-6, IL-8, IL-1β, TNFα) as well as release of antimicrobial peptide(s), such as defensins (Libby *et al.*, 2008; Eade *et al.*, 2012).

It is now appreciated that commensal bacteria and mucosal immune pathways are in continuous crosstalk. Bacteria actively shape host immune cell education and thereby support immunosurveillance of opportunistic infections at mucosal sites. At the endometrium, epithelial cells are the first line physical barrier between microbial and immune cell compartments. This interaction is also mediated by recognition on the part of both epithelial and immune cells of metabolites derived from the microbiome, hence suggesting an important role for these molecules. Dynamic changes in the local milieu due to cycle phase or pregnancy are likely to further influence these immune pathways. In this study, we profiled the endometrial microbiome in a cohort of women undergoing ART with known outcomes. We also exploited use of an *in vitro* model of receptivity and implantation to investigate the impact that microbial-derived metabolites might have on endometrial immune, receptivity and decidualization factors.

## Materials and methods

### Patient recruitment

**Figure 1.**
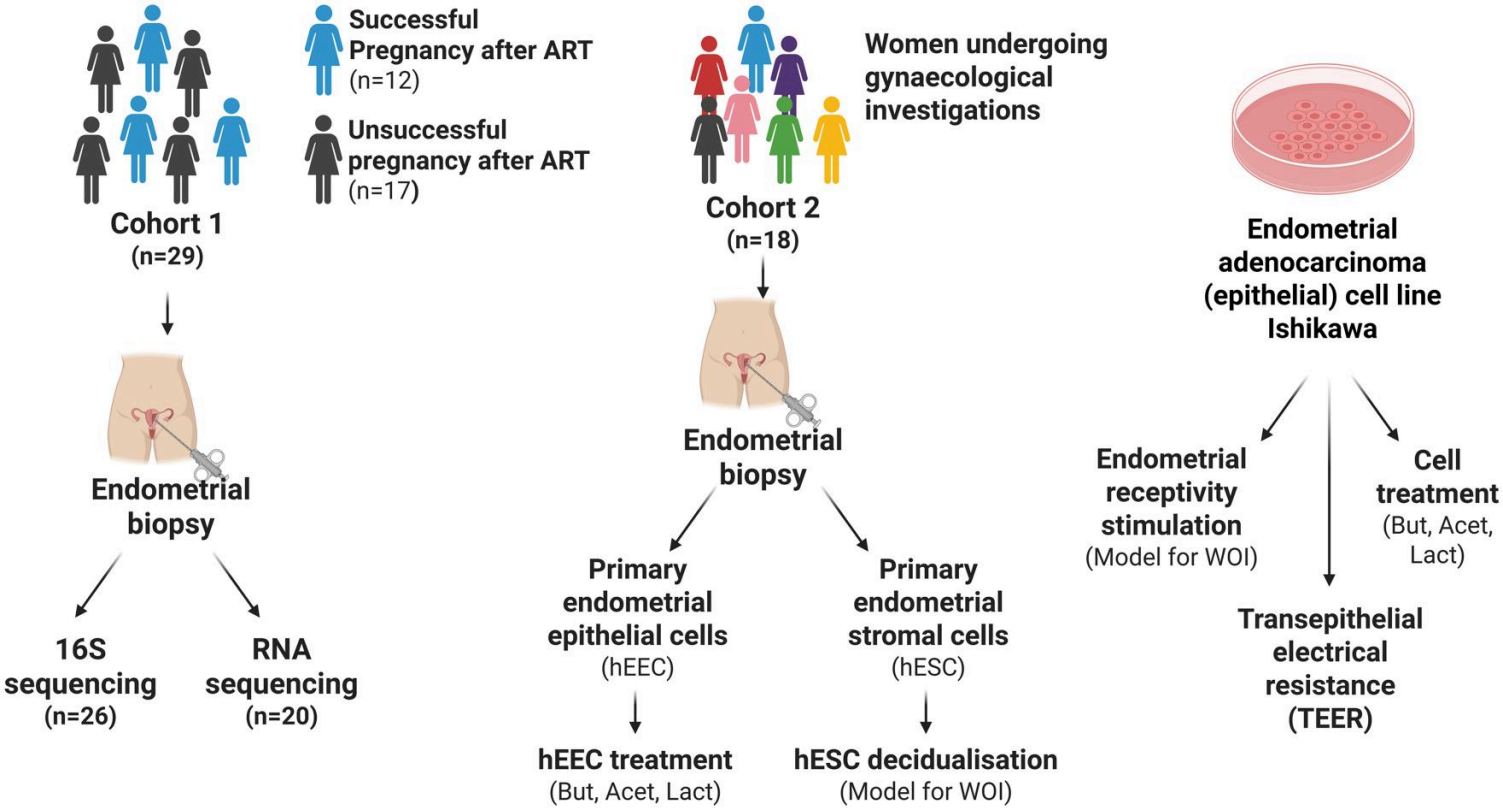
**Cohort recruited and experimental setup.** In this study, two cohorts of women were recruited. Cohort 1 comprised women undergoing ART (n = 29) from which endometrial biopsies were collected at day LH + 7. The endometrial biopsies were used to perform 16S-sequencing of microbial DNA and transcriptomic analysis with RNA-seq. The results from both these approaches were retrospectively correlated with the pregnancy outcome (12 successful pregnancies and 17 unsuccessful pregnancies). Endometrial biopsies were obtained from cohort 2 (n = 18) which comprised women undergoing gynaecological investigations. From these endometrial biopsies, primary human endometrial epithelial cells (hEECs) or primary human endometrial stromal cells (hESCs) were obtained and subjected to treatment with butyrate, acetate or lactate or subjected to *in vitro* decidualization, respectively. The endometrial epithelial tumoral cell model Ishikawa cells were used for treatments with butyrate, acetate or lactate (data not shown), transepithelial electrical resistance measurements or treated with progesterone for mimicking the window of implantation (WOI). ART, assisted reproductive technology; hEEC, primary human endometrial epithelial cells; hESCs, primary human endometrial stromal cells; But, butyrate; Acet, acetate; Lac, lactate; WOI, window of implantation; TEER, transepithelial electrical resistance.

Nulliparous women with unexplained infertility (Zegers-Hochschild *et al.*, 2017), who underwent ART at a hospital-affiliated fertility clinic between October 2016 and February 2018, were asked to allow the use of the endometrial scratch material for research (Fig. 1). Inclusion criteria included: age <38 years, no previous pregnancy (including no miscarriage), regular menstrual cycles (25–35 days), a normal transvaginal ultrasound scan, normal test of tubal patency, normal semen analysis in the partner, and no steroid hormone use within the preceding 3 months. Exclusion criteria included smoking, systemic disease, known cases of endometriosis, regular medication use and body mass index (BMI) ≥30 kg/m^2^. The characteristics of this cohort, here named cohort 1 (n = 29), have been previously published (Crosby *et al.*, 2020). The women in this cohort were matched for parity and embryo quality. The embryos transferred were not genetically tested for aneuploidies, however, the participants all received a single embryo transfer (SET) of a blastocysts of good to top quality.

A second cohort (cohort 2) of women (n = 18), recruited between February 2021 and October 2023, donated endometrial biopsies from which primary human endometrial and stromal cells could be isolated (Fig. 1). These were women of reproductive age who attended the National Maternity Hospital for gynaecological investigations due to pain, infertility or endometriosis.

Ethical approval for the study was granted by the National Maternity Hospital Ethics Research Committee (EC27.2016 and EC19.2018) and informed consent was obtained from participants prior to sample collection.

### 16S sequencing

Approximately 100–200 mg of endometrial tissue from cohort 1 (n = 26) was used to extract bacterial DNA using QIAmp UCP Pathogen Mini kit (QIAGEN, 50214) and with Pathogen Lysis tubes size S (QIAGEN, 19091), as already published (Leiby *et al.*, 2018). Negative contamination controls included theatre room air, glove swabs and water samples. A positive control consisting of a vaginal microbiome genome mix (NGS standard MSA-2007, ATCC) was also used.

The sequencing featured amplification of the variable regions V3–V4 of the 16S ribosomal RNA subunit gene. Primers were removed prior to filtering, denoizing and read merging and Amplicon Sequence Variants (ASV) were generated using DADA 2 package in R. ASVs were assigned to genus level using a naive Bayesian classifier method against the SILVA database. This resulted in a matrix of normalized reads associated with each bacterial genus. From this, relative abundance was calculated using the number of reads for each genus against the total number of reads within the same sample. Alpha-diversity was measured using both Shannon (which estimates the diversity of microbial species within each sample) and Simpson (which estimates the richness and evenness of species in a community) indexes calculation in R. The beta diversity between the two groups measured as the average steepness (z) of the species area curve in the Arrhenius model (S=cXz). Correlations were computed in GraphPad prism v8.0.1.

### RNA-sequencing analysis

Endometrial tissue for RNAseq was obtained from 20 women undergoing ART from cohort 1, of whom n = 9 became pregnant and n = 11 did not become pregnant. This study has been described previously (Crosby *et al.*, 2020) and data can be found at GSE144895.

### Primary endometrial epithelial and stromal cell isolation, culture and treatment

Endometrial tissue obtained with a Cornier pipelle was placed into transport medium: Hanks’ balanced salt solution (HBSS) supplemented with 100 U/ml penicillin–streptomycin, 2.5 μg/ml (corresponding to 5% vol/vol) Amphotericin B and 5% vol/vol charcoal-stripped foetal bovine serum (FBS). A single cell suspension was generated using previously described protocols (Chen and Roan, 2015; Thiruchelvam *et al.*, 2016). After washing with transport medium and mincing finely with a scalpel, biopsies were digested for 25 min in a shaking incubator at 37 °C at 150 rpm with the following digestion media: RPMI 1640, 20 mM HEPES, 1% vol/vol charcoal-stripped FBS, 1% vol/vol bovine serum albumin, 0.5 mg/ml Collagenase IV and 35 U/ml DNAse I. After inactivating with HBSS supplemented with 10% vol/vol charcoal-stripped FBS, a single cell suspension was obtained by filtering the cells with a 70 μm cell strainer and then endometrial stromal (hESCs) and epithelial (hEECs) cells were further separated through a 40 μm cell strainer and grown in complete growing medium: Keratinocytes Serum Free Media supplemented with 0.5% vol/vol of Penicillin–Streptomycin and Amphotericin B, 5% charcoal-stripped FBS and growing supplements (recombinant Epidermal Growth Factor, rEGF, 0.2 ng/ml and Bovine Pituitary Extract, BPE, 30 μg/ml).

Stimulation of hEECs with a titration of 0.5 mM, 2 mM and 8 mM butyrate, acetate or lactate (Sigma-Aldrich, UK, cat numbers B5887-250MG, S5636-500G and 1614308, respectively) was carried out for 6 h and 24 h using complete growing media. Supernatants were collected and cells were lysed in TRIzol™ Reagent for RNA extraction.

### Immortalized cell line culture and treatment

The human endometrial adenocarcinoma cell line Ishikawa (catalogue number 99040201-1VL) was purchased from the European Collection of Authenticated Cell Cultures (ECACC) repository. This tumoural cell line resembles the characteristics of endometrial epithelial cells and is widely used as a model for endometrial epithelium and for embryo implantation (Lessey *et al.*, 1996; Castelbaum *et al.*, 1997; Hannan *et al.*, 2010). This cellular model was chosen to perform stimulations alongside the primary hEECs and to perform complex experiments, for which the availability of an actively replicating cell type was beneficial.

Cells were grown in minimum essential media (MEM) supplemented with 5% vol/vol FBS, non-essential amino acids 1% vol/vol and penicillin–streptomycin 1% vol/vol (corresponding to 50 U/ml).

Ishikawa cells were stimulated with a titration of 0.5 mM, 2 mM and 8 mM butyrate, acetate or lactate (Sigma-Aldrich, UK) and the supernatant and RNA, lysed in TRIzol™ Reagent, were collected 6 h and 24 h after the stimulation.

### In vitro models of the WOI: hormonal treatment to induce endometrial receptivity

Decidualization of primary hESCs was performed as previously reported (Gibson *et al.*, 2018). Cells were treated with decidualization media (growth media supplemented with 1 μM progesterone and 0.1 mg/ml 8-Br-cAMP) in the presence or absence of either 2 mM sodium butyrate, 2 mM sodium acetate or 2 mM sodium Lactate (Sigma-Aldrich). The treatment was carried out for 1, 2, 4, and 6 days, and treatment media was replaced every 2 days. At the indicated timepoint, cells were lysed in TRIzol™ Reagent for RNA extraction.

Ishikawa cells have been reported to replicate the changes induced in the endometrium on treatment with sex hormones (Lessey *et al.*, 1996; Castelbaum *et al.*, 1997). Before the treatment, growth media was removed, and the cells were washed in PBS. Treatment was carried out in MEM media with the same formulation as the growth media, except for supplementation with 5% vol/vol charcoal-stripped FBS. Cells were treated with 1 μM Progesterone and 0.01 μM β-estradiol to induce epithelial receptivity, in the presence or absence of 2 mM sodium butyrate, acetate or lactate. The treatment was carried out for 1, 2, 3, and 4 days when cells were lysed in TRIzol™ Reagent; treatment media was replaced every 2 days.

### TEER measurements

For TEER measurements, Ishikawa cells were cultured on 0.33 cm^2^ transwell inserts. After seeding, TEER was measured daily using a voltohmeter (EVOM™, World Precision Instruments) to allow establishment of a polarized epithelial barrier. Once cells reached a raw measure of 1000 Ω per transwell (300 Ω cm^2^), they were stimulated with either 2 mM and 8 mM butyrate or 0.1 μM β-estradiol. TEER was measured daily up to 3 days after stimulation, media was collected from both apical and basolateral compartments, and the cells were lysed in TRIzol™ Reagent for RNA extraction.

### Viability assays

Viability assays were performed on cells seeded in triplicate in 96-well plates. Treatment volumes were adapted to ensure a final volume of 100 μl/well. A triplicate containing 100 μl/well of media only, without cells, served as a background media control. Two hours before harvesting, 10% vol/vol alamarBlue™ Cell Viability Reagent (Invitrogen) was added to each well and incubation was continued at 37 °C. The cell viability was assessed by measuring the absorbance of converted resorufin at 570 and 600 nm. The relative viability compared to the control was calculated.

Measurement of secreted lactate dehydrogenase (LDH) with CyQUANT™ LDH Cytotoxicity Assay (Invitrogen) in the apical and basal media, after the treatment of Ishikawa cells in transwells with either 2 mM and 8 mM butyrate or 0.1 μM β-Estradiol, was used to measure viability, following manufacturer’s instruction. Additionally, 10× lysis buffer (provided in the kit) was added to an untreated well to obtain the maximal LDH release measure which was used to calculate the relative percentage cytotoxicity measured with the absorbance of 490 and 680 nm.

### RNA extraction and qPCR

RNA extraction was performed using TRIzol™ reagent according to the manufacturer’s instructions and retrotranscribed to produce cDNA. PowerUp™ SYBR™ Green Master Mix was used to perform the qPCR according to the manufacturer’s guidelines using a QuantStudio5 qPCR machine. The primers used for the study are described in Supplementary Table S1.

### Immunohistochemical staining

To confirm the separation of hEECs and hESCs in the method used to obtain primary cell cultures, cells were plated on Millicell EZ slides (Merck-millipore, catalogue number PEZGS0816) and once confluent, washed with PBS and fixed for 10 min with ice-cold methanol. After another wash and blocking of endogenous peroxidases with 3% hydrogen peroxide incubation for 20 min, cells were left for 1 h with casein block (Vector Lab Supplies). The slides were washed once, and primary antibodies against cytokeratin (Dako, M3515), CD10 (Abcam, ab126593) or isotype control were incubated overnight at 4 °C. After three washes in PBS, the slides were incubated for 30 min with EnVision Detection Systems Peroxidase/DAB Kit (Dako, K5007). Freshly prepared DAB was added to the cells and incubated until colourimetric change was observed and then counterstained with haematoxylin. After a first wash with water, cells were washed once more with PBS and then air dried before mounting on coverslips using Pertex Mounting medium (Pioneer Research Medicals, PRC/R/750). Pictures were obtained using an Olympus BX51 upright microscope.

### Statistical analysis

GraphPad Prism v8.0.1 was used for statistical analysis. Normal distribution of data was assessed using Shapiro–Wilk test to determine whether parametric or non-parametric tests should be used. Unpaired *t*-tests were applied to the 16S sample cohort characteristics (pregnant vs not pregnant). For *in vitro* assay outcomes, two-way ANOVA tests were used to analyse differences, with Dunnett or Sidak correction for multiple testing, as indicated. Mixed-effects model fitting test was applied where two-way ANOVA could not be used due to missing values.

Linear regression analysis was applied to the CPM values obtained from RNA-sequencing and the 16S-sequencing derived data. The analysis was performed on the whole dataset or on only the pregnant or not pregnant outcome subgroups.

## Results

### Women who did not become pregnant after ART show a more diverse microbiome and increased endometrial receptivity markers

Overall, 47 women were included in the study (Table 1). As previously published (Crosby *et al.*, 2020), cohort 1 (n = 29) were all nulliparous women with unexplained infertility. Endometrial tissue biopsies were collected in the mid-luteal phase (LH + 7). In the subsequent menstrual cycle, study participants underwent a day five SET of a top or good-quality blastocyst. Subjects were then grouped by ART outcome, i.e. those who did (n = 12) or did not (n = 17) become pregnant (Crosby *et al.*, 2020). Since we had previously observed increased levels of the pro-inflammatory cytokine IL-17A in the non-pregnant group, and because IL-17A is known to control bacterial infections, we wondered whether these women showed a difference in their endometrial microbiome. A second cohort (cohort 2) of women attending surgical laparoscopies was recruited to isolate primary hEECs and hESCs.

**Table 1.** Patient characteristics of study cohorts.

Characteristics	Cohort 1 (n = 29)	Cohort 2 (n = 18)
Age (years)	35.2 ± 1.5	36.5 ± 8.8
BMI, kg/m^2^	23.1 ± 2.7	29.9 ± 6.1
Parity (number of live births)	0 ± 0	1.3 ± 1.4
Menstrual cycle length		
Regular (21–35 days)	28 (96.6)	11 (61.1)
Irregular (<21 days, >35 days)	1 (3.4)	5 (27.8)
N/A^a^	0 (0)	2 (11.1)
Menstrual phase at collection^b^		
Menstruation	0 (0)	3 (16.7)
Proliferative	0 (0)	2 (11.1)
Ovulatory	0 (0)	2 (11.1)
Secretory	29 (100)^c^	6 (33.3)
N/A^a^	0 (0)	5 (27.8)
Diagnosis of infertility		
Yes	29 (100)	8 (44.4)
No	0 (0)	10 (55.6)
Diagnosis of endometriosis		
Yes	0 (0)	3 (16.7)
No	29 (100)	15 (83.3)
Pain		
Yes	0 (0)	9 (50)
No	29 (100)	7 (38.9)
N/A^a^	0 (0)	2 (11.1)
Hormonal treatment		
Yes	0 (0)	6 (33.3)
No	29 (100)	6 (33.3)
N/A^a^	0 (0)	6 (33.3)

Values presented as mean±SD or as n (%).

a N/A assigned when data were not available for that donor.

b Menstrual phase was annotated as follows: cycle Days 1–7= Menstruation, Days 8–12= Proliferative, Days 13–15= Ovulation, Days 16–28 = Secretory.

c Cohort 1: biopsy samples collected at defined stage of menstrual cycle (LH + 7).

**Figure 2.**
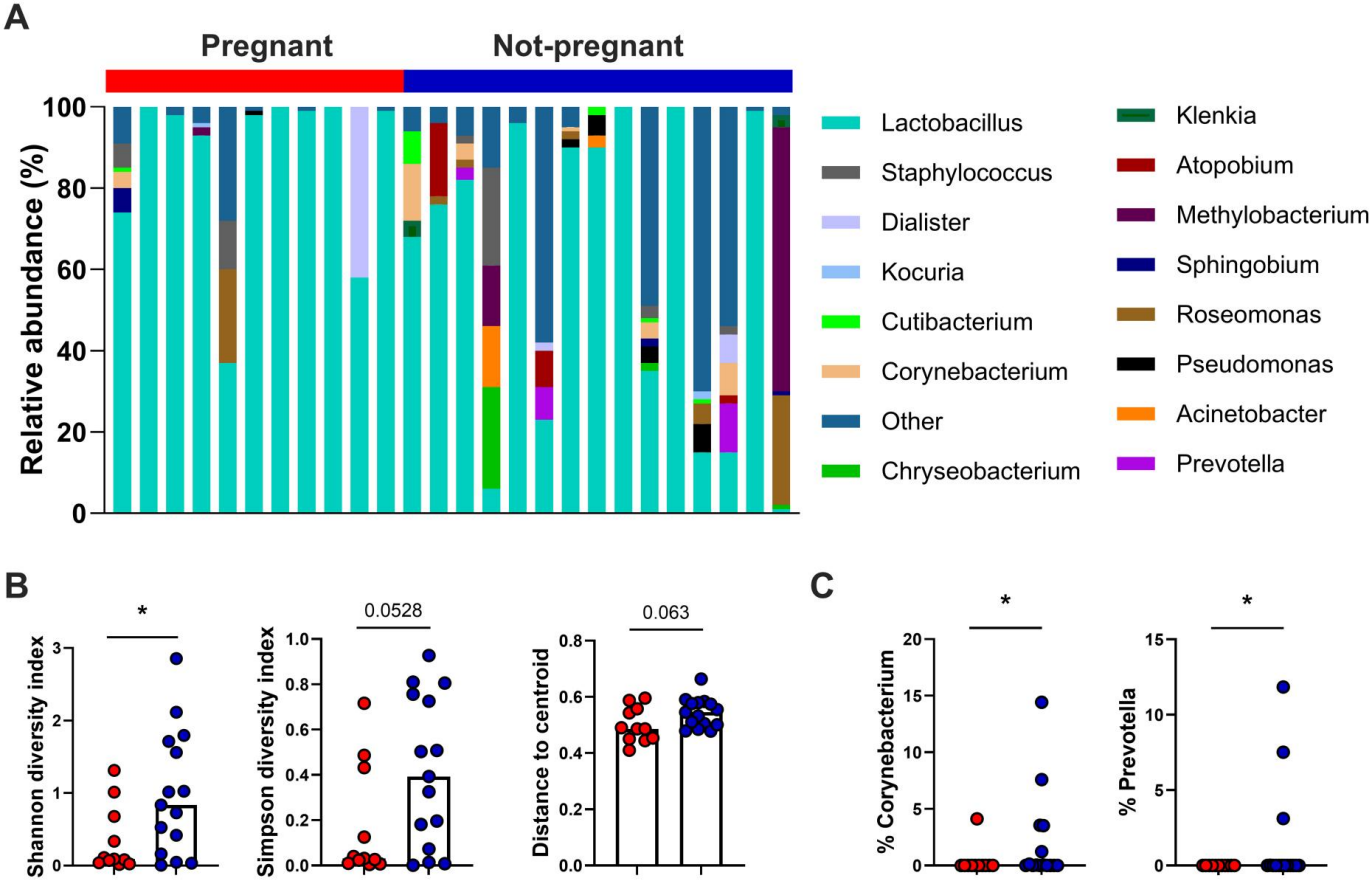
**Women who failed to establish a successful pregnancy after ART have more diverse endometrial microbiome.** The counts obtained from 16S sequencing of bacterial DNA derived from endometrial biopsies (n = 26) of women who become pregnant (n = 11) or did not (n = 15) were used to calculate the relative abundance of bacteria, expressed as percentage of abundance. The counts matrix was also used to calculate the diversity in microbiome composition and to generate the heatmap. (**A**) Plot of the values of relative abundance for each bacterial genus in each sample. ART outcome is color-coded with red for pregnant and blue for not pregnant. (**B**) Shannon, Simpson and beta-diversity indices plotted by ART outcome. Statistical tests applied: unpaired *t*-test with *P < 0.05. (**C**) The percentage abundance of Corynebacterium and Prevotella was plotted by pregnancy outcome. Statistical tests applied: Mann–Whitney non-parametric test, with *P < 0.05.

To investigate endometrial microbial communities, 16S sequencing was performed on bacterial DNA isolated from endometrial biopsies collected from women in cohort 1 (n = 11 pregnant and n = 15 non-pregnant). Women who had a successful ART cycle and became pregnant had a microbiome dominated by *Lactobacillus* spp. with a low abundance of other species, whereas women who did not become pregnant showed increased abundance of other bacterial species, contributing to a significant increase in the diversity index in their microbiome (Fig. 2A and B). Particularly, abundance of *Corynebacterium* and *Prevotella* spp. was significantly increased in the non-pregnant group (Fig. 2C).

**Figure 3.**
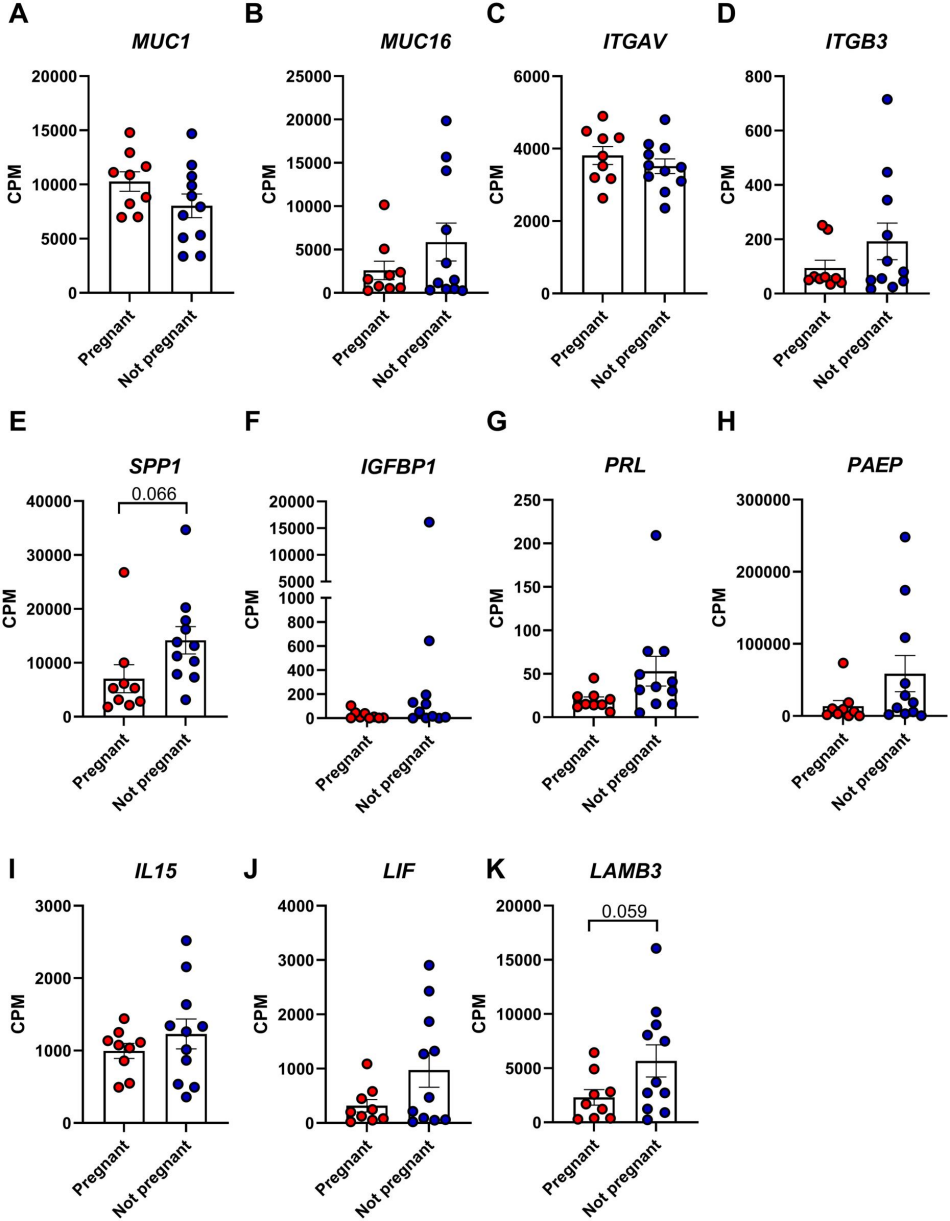
**Women with unexplained infertility who did not become pregnant after ART have higher levels of epithelial receptivity and stromal decidualization markers than women who become pregnant.** The counts per million obtained from bulk RNA sequencing of endometrial biopsies (n = 20) of women who were pregnant (n = 9) or non-pregnant (n = 11) after ART were used to investigate the expression levels of markers associated with endometrial receptivity. (**A–E**) Plot of genes linked to epithelial receptivity, whereas (**F–K**) show genes expressed by the decidual compartment during the opening of the window of implantation. Statistical tests applied: unpaired *t*-test with Welch’s correction. CPM, counts per million.

Given the difference in ART cycle outcomes in these women, we also wished to establish whether there were any differences in endometrial receptivity. We re-interrogated bulk RNA-seq data generated from endometrial biopsy transcriptomes from 20 women of cohort 1 (Crosby *et al.*, 2020). Intriguingly, women who did not become pregnant showed increased expression of several receptivity and decidualization markers, such as ITGB3, SPP1, PRL and LAMB3 (Fig. 3).

### Butyrate, a common microbiome-derived metabolite changes endometrial receptivity marker expression

Due to the proximity between the local microbiome and the endometrial specialized epithelium and stromal layers, we wished to ascertain whether changes in the microbiome could impact endometrial receptivity markers.

**Figure 4.**
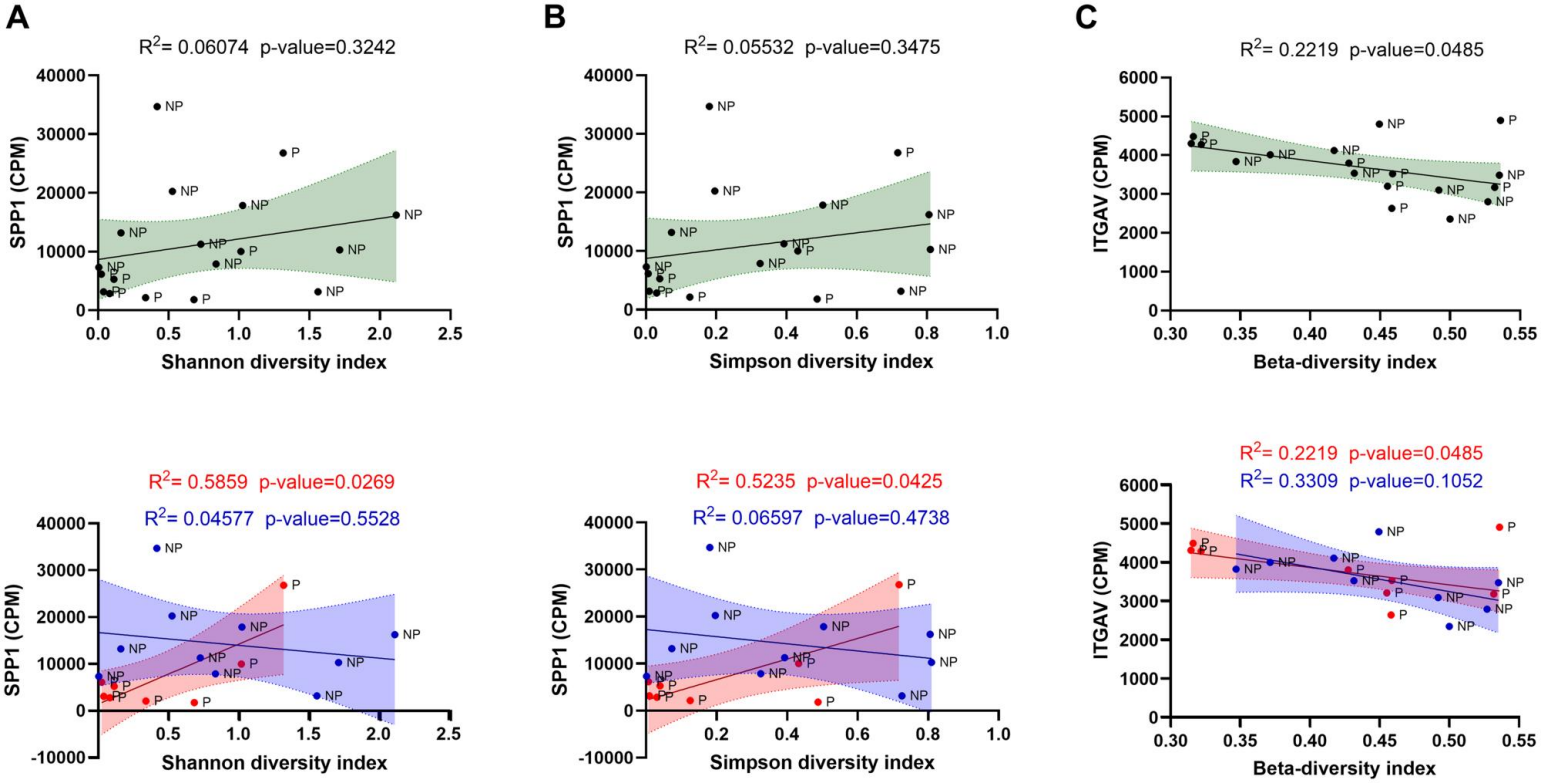
**Endometrial microbiome diversity of women with unexplained infertility who did not become pregnant correlates with receptivity marker expression.** (**A**) Linear regression of SPP1 expression against Shannon diversity index, for the combined cohort in the upper section and split between pregnant (red) or not pregnant (blue) in the lower section. (**B**) Linear regression of SPP1 expression and Simpson diversity index, (**C**) Linear regression of ITGAV expression and the beta-diversity index. The statistical tests applied for the diversity indices were two-tailed parametric unpaired *t*-tests, with *P < 0.05; the statistical test applied for Corynebacterium and Prevotella % abundance was a non-parametric Mann–Whitney test, with *P < 0.05. CPM, counts per million.

We performed linear regression analysis to identify any correlation between the microbiome diversity and the receptivity marker expression. We performed this test for the original discovery cohort (n = 20: pregnant n = 9 or not pregnant n = 11) and calculated the regression for pregnant or non-pregnant women. Using this approach, we identified significant correlations between the microbiome diversity indexes and expression levels of SPP1 and ITGAV receptivity markers expression (Fig. 4, Supplementary Tables S2 and S3). Intriguingly, both the Shannon and Simpson diversity indexes correlated positively with SPP1 expression only in women who did become pregnant, whereas in the non-pregnant group a negative correlation was observed (Fig. 4A and B). ITGAV expression was negatively correlated with the beta diversity from the entire cohort, whereas there was no correlation within the pregnant or non-pregnant groups (Fig. 4C). Linear regression of the receptivity markers and the abundance of either *Lactobacillus* spp. and *Prevotella* spp. did not result in any significant correlation (Supplementary Table S4). Similarly, correlation analysis between immune mediators and antimicrobial peptides against either the diversity indexes (Supplementary Table S5) or abundance of *Lactobacillus* spp. and *Prevotella* spp. (Supplementary Table S6) did not show any significant correlation.

**Figure 5.**
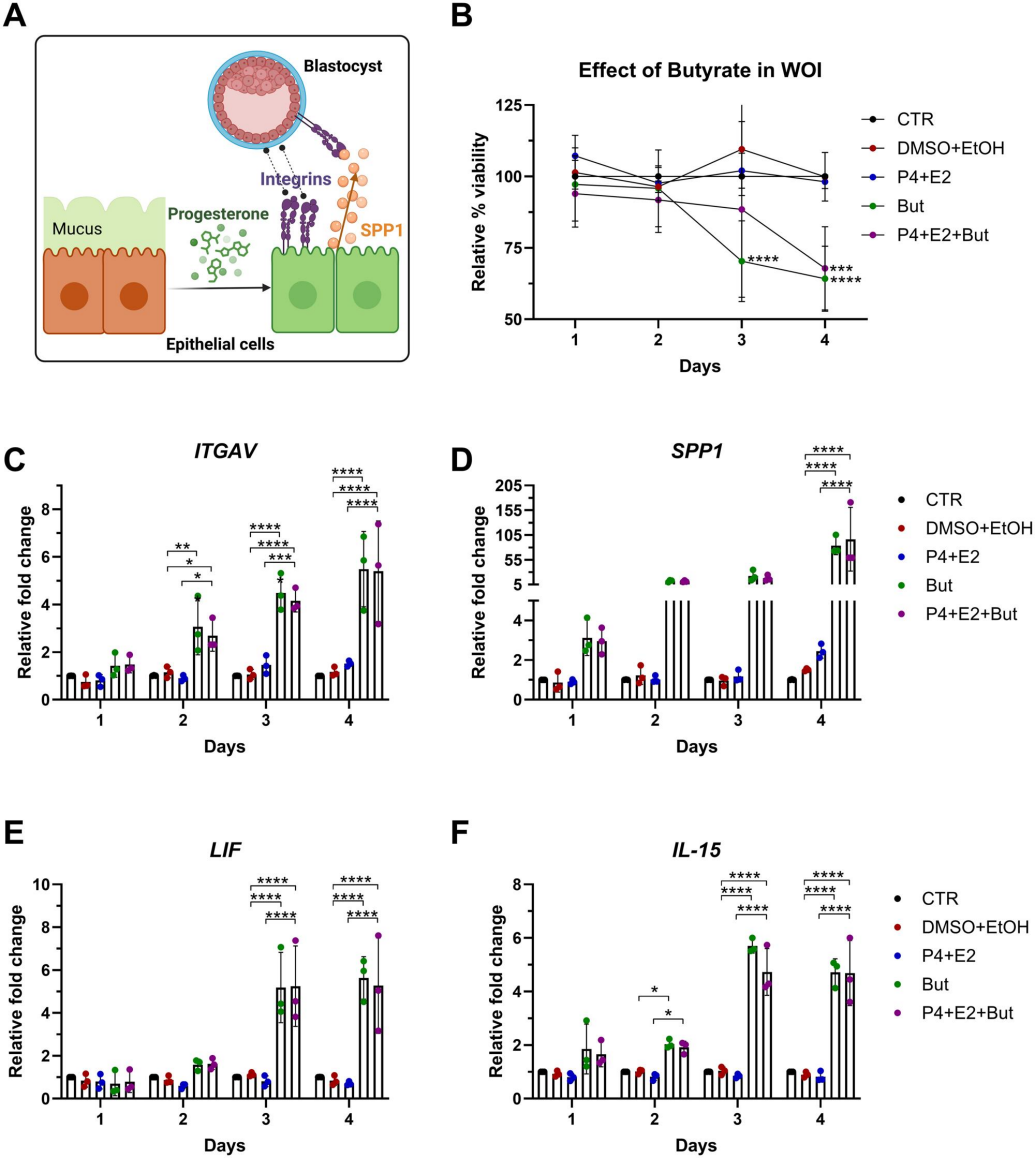
**Butyrate enhances the epithelial expression of ITGAV and SPP1, known markers associated with endometrial receptivity, as well as LIF and IL-15, markers expressed by decidual cells.** Ishikawa cells were treated for up to 4 days with P4 (1 μM) and E2 (10 nM) or with equal amounts of dissolving medium controls (DMSO or Ethanol, respectively), with or without 2 mM butyrate (But). The treatment media was replaced every 2 days. (**A**) Overview of the processes induced by progesterone during endometrial epithelial receptivity. (**B**) Cell viability was assessed by adding to the growing media 10% vol/vol Alamar Blue and reading conversion of resazurin in live cells. Calculation of cell viability was performed using the control cells as reference. (**C–F**) RNA was extracted and expression of ITGAV (C), SPP1 (D), LIF (E) and IL-15 (F) was assessed with qPCR using RPLP0 as reference gene. N = 3. Statistical test applied: Two-way ANOVA with Tukey correction. *P < 0.05; **P < 0.002; ***P < 0.0002; ****P < 0.0001. ITGAV, integrin subunit alpha V; SPP1, secreted phosphoprotein 1; LIF, leukaemia inhibitory factor; IL-15, interleukin-15; But, butyrate; DMSO, dimethyl sulfoxide; E2, oestradiol; EtOH, ethanol; P4, progesterone.

**Figure 6.**
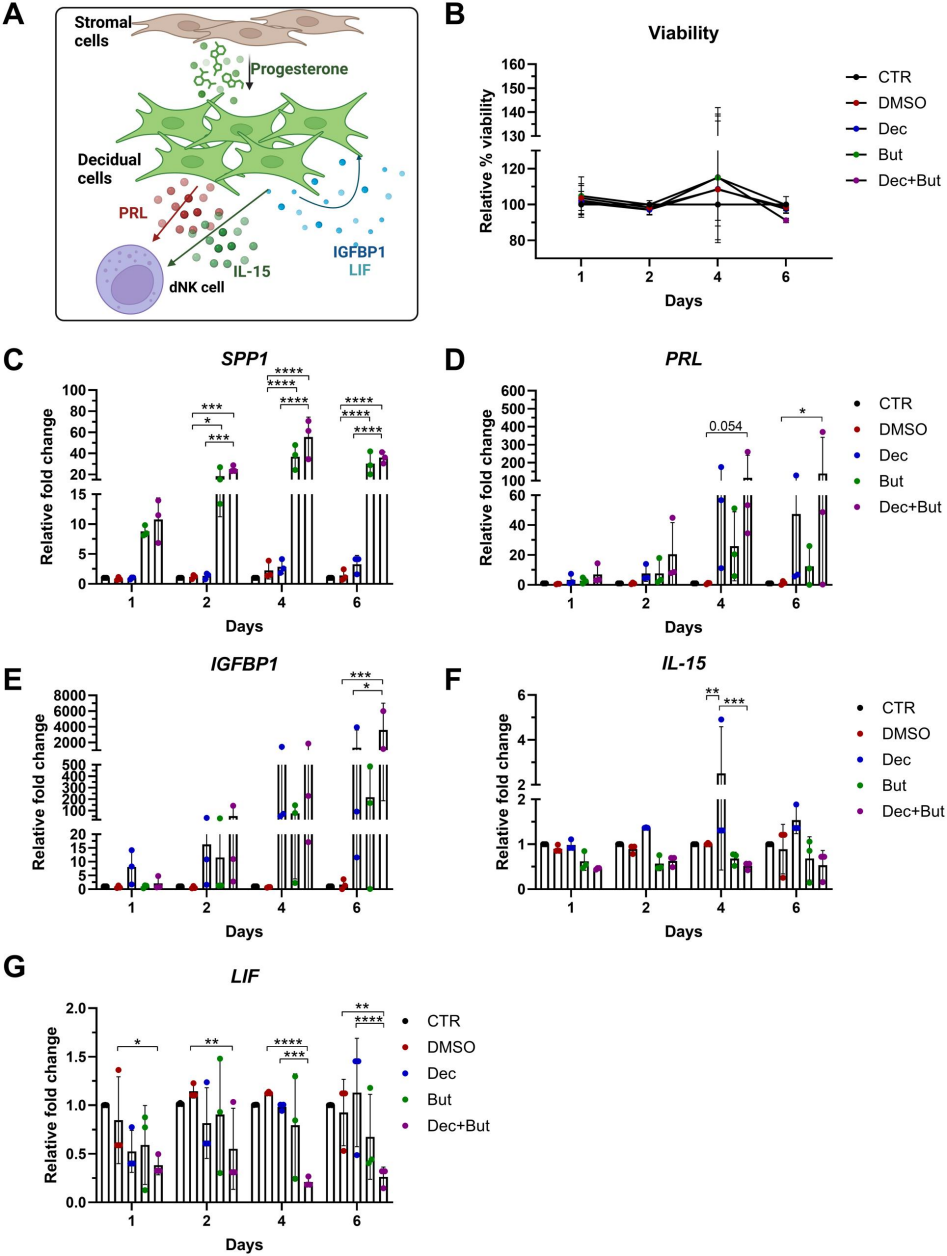
**Butyrate enhances stromal decidualization markers SPP1, PRL and IGFBP1 but decreases LIF and IL-15 in stroma.** (**A**) Overview of the decidualization process induced in endometrial stromal cells by progesterone. Human endometrial stromal cells were isolated from endometrial biopsies. Cells were treated for up to 6 days with decidualization media (P4, 1 μM, and 8-Br-cAMP, 0.1 mg/ml) or with equal amounts of dissolving medium controls (DMSO), in the presence or absence of 2 mM Butyrate. Treatment media was refreshed every 2 days. (**B**) Cell viability was assessed by adding 10% vol/vol Alamar Blue and recording resazurin conversion at each timepoint and then calculate the relative % viability compared with control cells. (**C–G**) RNA was extracted and qPCR was performed using RPLP0 as housekeeping reference gene. Shown is relative gene expression of (**C**) SPP1, (**D**) PRL, (**E**) IGFBP1, (**F**) IL-15 and, (**G**) LIF. N = 3. Statistical test applied: Two-way ANOVA with Tukey correction. *P < 0.05; **P < 0.002; ***P < 0.0002; ****P < 0.0001. SPP1, secreted phosphoprotein 1; PRL, prolactin; IGFBP1, insulin-like growth factor-binding protein-1; LIF, leukaemia inhibitory factor; IL-15, interleukin-15; RPLPO, ribosomal protein lateral stalk subunit P0 (60S subunit); But, butyrate; Dec, decidualization media; DMSO, dimethyl sulfoxide; EtOH, ethanol.

Given the association between microbiome diversity and receptivity marker expression in endometrial tissue, we wished to determine whether microbial-derived metabolites could affect endometrial receptivity marker expression *in vitro*. We developed and tested *in vitro* models for epithelial receptivity and stromal decidualization in the presence of bacterial-derived metabolites such as acetate and butyrate, chosen to represent the dysbiotic microbiome, or lactate, which represents the healthy *Lactobacillus* spp.-dominated microbiome. We found that butyrate (Figs 5 and 6), but not lactate or acetate (Supplementary Figs S1, S2, and S3), greatly increased epithelial receptivity and stromal decidualization marker expression. In the context of endometrial receptivity, opening of the WOI is associated with increased expression on endometrial epithelial cells of factors that both attract (SPP1) and interact with the implanting embryo (ITGAV). In particular, butyrate significantly impacted the expression of ITGAV and SPP1 in the endometrial epithelial cell line Ishikawa when treated with progesterone to simulate the changes induced by the receptivity window (Fig. 5C and D). Furthermore, butyrate induced the expression of IL-15 and LIF by Ishikawa epithelial cells, markers that are induced in the decidualized endometrium during the WOI (Fig. 5E and F).

Some of these results were replicated in primary hESCs cultures, derived from tissue biopsies. Progesterone treatment was used to induce decidualization *in vitro*. Known decidualization markers include PRL and IGFBP1, and SPP1 is also expressed by decidual cells to guide correct embryo implantation. LIF and IL-15 are thought to mediate the balance between inflammation and tolerance in immune cells present in the decidua. Butyrate significantly induced the expression of SPP1 in stromal decidual cells derived from hESCs (Fig. 6C). Other known stromal decidualization markers, such as PRL and IGFBP1, were shown to be induced by the decidualization media alone or in conjunction with butyrate (Fig. 6D and E). Notably, at Days 4 and 6, cells treated with butyrate alone displayed increased PRL and IGFBP1 expression (Fig. 6D and E), possibly contributing to their enhanced expression by Day 6 in butyrate-supplemented decidualization media compared to decidualization media alone. Conversely, IL-15 and LIF were significantly downregulated in decidualized stromal cells treated with butyrate, suggesting that butyrate could contribute to dysregulated inflammatory signalling in the stromal compartment (Fig. 6F and G).

Interestingly, butyrate was shown to induce cell death in the epithelial cell receptivity model (Fig. 5B), but not in the hESCs decidualized stromal cell model (Fig. 6B), possibly reflecting differential susceptibility of endometrial cellular compartments to the microbial-derived metabolite. Despite the cell death induced in the epithelial receptivity model, an increase in ITGAV and SPP1 expression was notable as early as Day 2, where the levels of cell death are comparable to the control or other stimulations.

These findings suggest important interactions between local microbiota ecostructure and endometrial receptivity. Microbial-derived metabolites, such as butyrate, may alter the expression of endometrial receptivity markers.

### Butyrate affects epithelial barrier integrity and activates pro-inflammatory signalling in endometrial epithelial cells

To understand how butyrate modifies endometrial receptivity, we assessed the effect of butyrate on epithelial layer integrity, the primary cellular contact for microbial-derived metabolite secretion at the endometrial mucosa.

**Figure 7.**
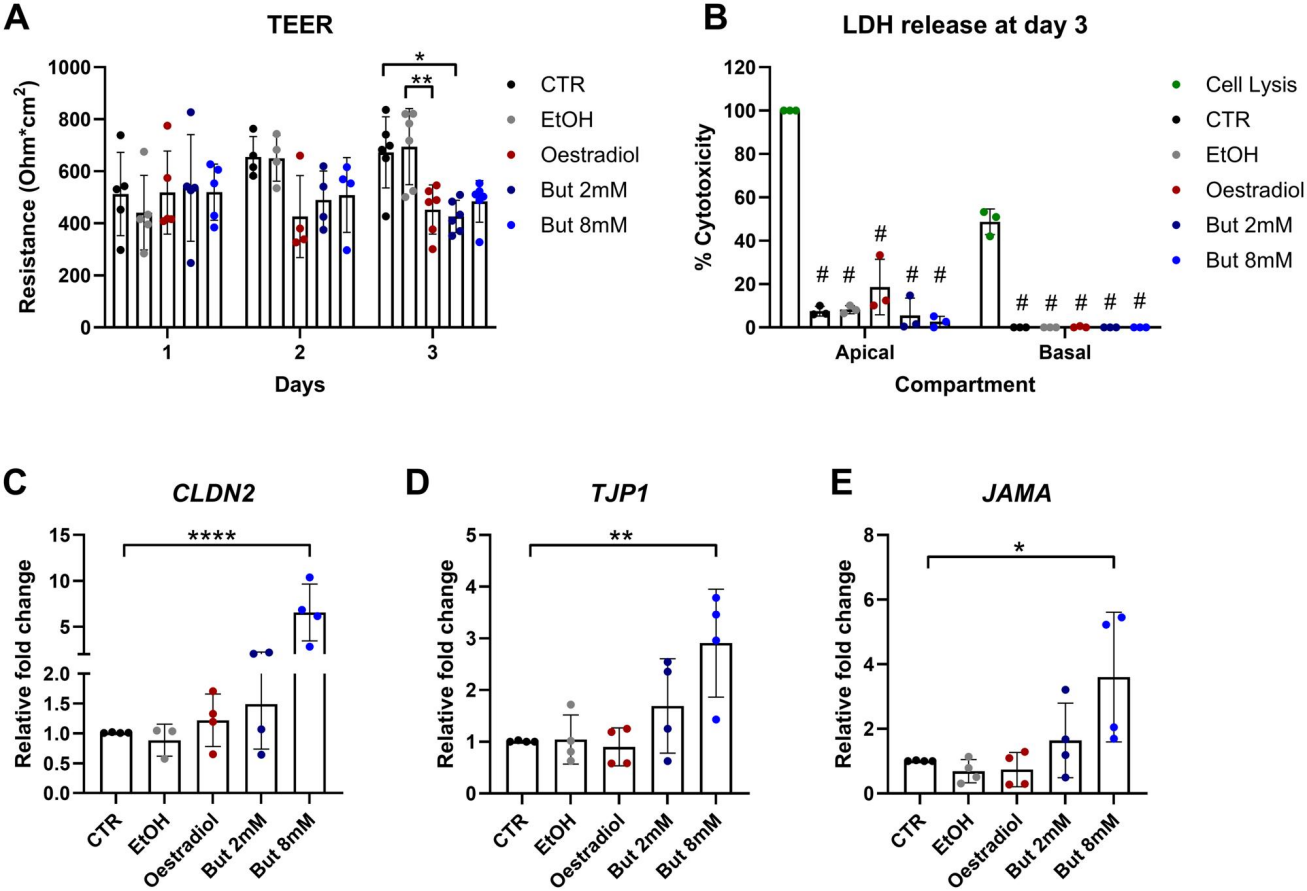
**Butyrate impairs epithelial integrity.** (**A**) Transepithelial/trans-endothelial electrical resistance (TEER) was measured in endometrial adenocarcinoma cells after 1, 2, or 3 days of butyrate (8 mM and 2 mM) or oestradiol stimulation. (**B**) Cell death was assessed by measuring LDH release in media obtained from the apical or basal compartment of the transwell system used to polarize the epithelial monolayer after 3 days of stimulation. (**C–E**) After 3 days of stimulation with oestradiol or butyrate, cells were lysed and RNA extracted to perform qPCR for tight junction and adhesion molecules encoding genes, such as CLDN2, TJP1 and JAMA. Statistical test applied: Two-way ANOVA with Tukey correction. For graphs (**A**) and (**C–E**). statistics are represented by: *P < 0.05; **P < 0.002; ****P < 0.0001. For graph (**B**), statistics are represented by # to summarize *P* < 0.0001 obtained by comparing against the maximal LDH release from lysis buffer. But, butyrate; CTR, control; EtOH, ethanol.

In the gut, butyrate is known for its beneficial role in maintaining barrier integrity, therefore, we hypothesized that butyrate could also affect the endometrial epithelial cell barrier function. Other studies (Wira *et al.*, 2010) have shown that oestradiol can impair barrier integrity. Thus, oestradiol was used as a reference to test TEER. With this approach, we were able to demonstrate that, in contrast to gut epithelial layer function, butyrate significantly decreased electric resistance of the epithelial barrier formed by endometrial epithelial cells, in a manner similar to the reduction observed with oestradiol (Fig. 7A). This effect was not due to increased cell death, tested by measuring LDH release from damaged plasma membranes after 3 days of butyrate stimulation (Fig. 7B). The alteration of the epithelial barrier was most likely due to tight junction loosening, as confirmed by the increase in expression of claudin 2 (CLDN2), which is typically expressed in leaky epithelia, as well as the increased expression of TJP1 and JAMA: two proteins involved in tight junction formation (Fig. 7C–E).

We next tested the effect of butyrate on epithelial cell pro-inflammatory activation. In the context of BV, SCFAs (such as butyrate or acetate), can induce inflammation in vaginal epithelial cells. We wondered if butyrate (or acetate or lactate), would show a similar effect in endometrial epithelial cells.

**Figure 8.**
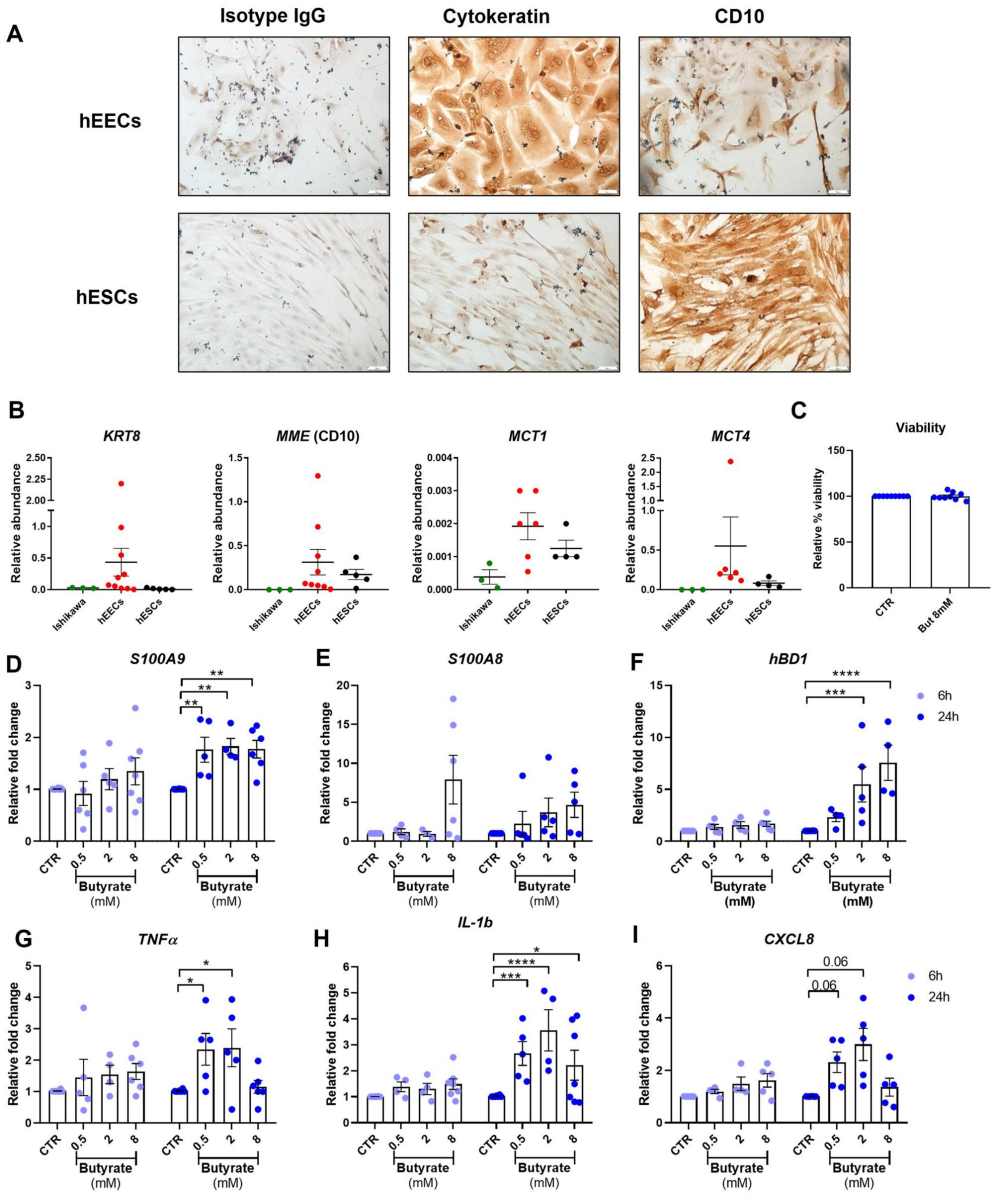
**Butyrate induces pro-inflammatory molecule expression in human endometrial epithelial cells (hEECs).** (**A, B**) hEECs and human endometrial stromal cells (hESCs) cells were isolated from endometrial biopsies. They were confirmed as epithelial or stromal cells by immunohistochemistry and tested by qPCR for the expression of known markers for endometrial epithelial (cytokeratin) or stromal cells (CD10), as well as the expression of the transporter needed to internalize butyrate, MCT1 and MCT4. (**C**) hEECs were treated for 24 h with 8 mM butyrate and cell viability was obtained with Alamar Blue viability assay. The relative % viability was calculated by using the reading of the control sample as reference. (**D–I**) hEECs were treated with a titration of butyrate (0.5, 2, and 8 mM) for 6 h and 24 h and RNA was extracted, and qPCR performed for assessing the changes in expression of (**D–F**). Antimicrobial peptides (S100A9, S100A8 and human β-defensin 1 hBD1) and of (**G–I**) inflammatory mediators IL1β, TNFα and CXCL8. RPLP0 gene was used as housekeeping reference for qPCR analysis. N ≥ 3. Statistical test applied: Mixed-effects model with Dunnett correction. But, butyrate; CTR, control; hEECs, human endometrial epithelial cells; hESCs, human endometrial stromal cells.

We established a culture of primary hEECs, obtained from endometrial biopsies collected from women (n = 18) undergoing laparoscopic investigations. We confirmed that this was a pure endometrial epithelial cell line by microscopical inspection with immunohistochemistry, and by testing the expression of cytokeratin 8 (Fig. 8A and B), as well as the presence of MCT1 and MCT4: the transporters needed for internalization of SCFAs. We then tested the effects of butyrate on antimicrobial peptides and inflammatory marker expression. hEECs responded to butyrate stimulation by significantly increasing the expression of antimicrobial peptides S100A8, S100A9 and human β-defensin 1 in a dose-dependent manner (Fig. 8D and E). These molecules are produced by epithelial cells during bacterial infection to fight the pathogen either through depleting essential metals needed for bacterial growth or by direct killing through inducing pores in microbial membranes. Furthermore, the S100A8/A9 dimer is also released during inflammation. We also measured the expression and secretion of other inflammatory mediators, in particular TNFα, IL1b and CXCL8. Butyrate was found to greatly induce an increase in the expression of all three inflammatory molecules. However, the effect observed did not follow a dose-dependent pattern but it either showed similar levels of expression between doses or a peak of highest expression with 2 mM butyrate (Fig. 8G–I). We also tested whether acetate or lactate had similar effects. Treatment of hEECs with a titration of either acetate or lactate did not change the expression of these markers (Supplementary Fig. S4). These results were also recapitulated in the Ishikawa epithelial cell line, where only butyrate induced inflammation and antimicrobial peptide expression (data not shown).

Thus, we confirmed that butyrate, but not acetate or lactate, can impair the endometrial epithelial barrier and also trigger the expression of antimicrobial peptides, cytokines and chemokines, which contribute to an increased pro-inflammatory activation in the endometrium.

## Discussion

We previously reported increased activation of the IL-17A pathway (Crosby *et al.*, 2020), a typical pro-inflammatory response to bacterial infection (McGeachy *et al.*, 2019), in endometrial tissue from women with unexplained infertility who failed to establish pregnancy after ART. In this follow-up study of the same cohort of women, we observed decreased abundance of *Lactobacillus* spp. in the women who did not become pregnant, and increased microbial diversity in their endometrial microbiomes.

Loss of *Lactobacillus* spp. predominance has been reported to negatively impact implantation and pregnancy rates (Franasiak *et al.*, 2016; Moreno *et al.*, 2016). In these previous studies, Gardnerella and Streptococcus abundance seemed to negatively impact pregnancy establishment and live birth rates (Moore *et al.*, 2002; Moreno *et al.*, 2016). Conversely, women presenting with a dysbiotic endometrial microbiome with 0% Lactobacillus content had pregnancy rates comparable to women harbouring an eubiotic Lactobacillus-dominated microbiome (Hashimoto and Kyono, 2019). The authors suggest that the presence of a particular species or set of species might have a bigger impact on pregnancy outcomes than a discriminatory Lactobacillus versus non-Lactobacillus microbiome at the genus level (Hashimoto and Kyono, 2019).

The ERA has been used extensively in the clinical ART setting to assess expression of markers associated with endometrial receptivity, to predict the opening of the WOI and to guide the timing of embryo transfer (Díaz-Gimeno *et al.*, 2011). During the endometrial cycle, the sex hormone progesterone induces maturation of epithelial and decidualization of stromal cells to express molecules that are involved in embryo implantation (Harper, 1992; Dunn *et al.*, 2003; Achache and Revel, 2006; Herington *et al.*, 2016). Here, we replicated the ERA with a view to testing the effect of microbiome-derived metabolites on receptivity marker expression. Timed endometrial biopsies were collected from our study cohort during the opening of the WOI, coinciding with Day 7 post-LH surge. RNA-seq gene expression data for known markers of endometrial receptivity showed increased expression in women who did not become pregnant in a subsequent cycle compared to the pregnant group. This finding suggests that fine-tuning of receptivity marker expression is fundamental for embryo implantation and successful pregnancy establishment. This has also been indicated in studies where the efficacy of the ERA in improving live birth rates among infertility populations has been questioned (Lensen *et al.*, 2021).

As the endometrial microbiome has been shown to impact female fertility (Moreno *et al.*, 2022), we wished to determine whether it could modulate endometrial receptivity marker expression. Linear regression analysis between the counts per million obtained from the RNA-seq and the readouts from the 16S sequencing showed that SPP1 and ITGAV, two molecules needed for the interaction and attraction of the embryo to the implantation site, negatively correlated with the diversity indexes in the group of women who did not become pregnant. Diversity indexes positively correlated with SPP1 expression in the endometrial samples from women who became pregnant, whereas a negative correlation was observed with ITGAV. To confirm this observation, we established *in vitro* models for replicating the changes induced by progesterone in epithelial and stromal cells during the receptivity window, as previously described (Lessey *et al.*, 1996; Gibson *et al.*, 2018). Supplementation of receptivity media with the microbial-derived SCFAs butyrate, acetate or lactate mimicked the high diversity microbiome (butyrate or acetate) or the *Lactobacillus* spp. dominated microbiome (lactate). Intriguingly, only butyrate, added either alone or to the receptivity media, caused a significant increase in the expression of receptivity markers in both the epithelial and stromal decidual cellular models. Furthermore, LIF and IL-15, two cytokines produced by decidual cells and known to interact with uterine natural killer cells (Mariee *et al.*, 2012; Wang *et al.*, 2021) were downregulated in cells treated with decidualization media and butyrate, suggesting that the effect of butyrate is to disrupt the tightly balanced expression of decidualization markers, leading to possible implantation failure. These results confirm that the microbiome can modulate receptivity marker expression through the secretion of microbial-derived metabolites such as butyrate. A direct comparison between the results from the *in vitro* models for the WOI and the results obtained from the RNA-seq expression of receptivity markers should be considered with caution, as these were two profoundly different models. Bulk RNA-seq results derive from an endometrial tissue sample consisting of a mixture of epithelial, stromal and immune cells which had also an interaction with the local microbiome, whereas the models *in vitro* consist of a singular cell type grown aseptically. Notably, however, the increased expression of receptivity markers in the non-pregnant group (Fig. 3) aligned with increased expression of epithelial and stromal decidualization markers (SPP1, PRL, ITGBP1) induced by butyrate (Figs 5 and 6), mimicking the dysbiosis presented in those women. Conversely, ITGAV showed similar levels between pregnant and not pregnant group, but its expression was enhanced by butyrate in the cultured epithelial receptivity model. We noted that ITGB3, which is known to interact with both ITGAV and SPP1 (Kang *et al.*, 2014), was increased in non-pregnant group. One possible explanation for conflicting ITGAV expression results might be related to the timing of tissue sampling.

SCFAs are mostly known for their anti-inflammatory role in the gut, where they are essential for healthy homeostasis, epithelial cell proliferation and barrier integrity (Kelly *et al.*, 2015; Park *et al.*, 2016). In that location, butyrate is particularly important for maintaining an immunosuppressive environment through inhibition of NFκB and epithelial barrier integrity promotion (Gaudier *et al.*, 2004; Thangaraju *et al.*, 2009). Conversely, in the context of BV, this molecule is involved in pro-inflammatory cytokine and antimicrobial peptide expression (Libby *et al.*, 2008; Eade *et al.*, 2012). Very little is known regarding the homeostatic concentration of SCFAs, and butyrate in particular, in the endometrium, although measurement of SCFAs in cervicovaginal mucus from healthy donors showed concentrations of ∼0–4 mM acetate and less than 1 mM butyrate (Aldunate *et al.*, 2015). We investigated the effect of butyrate on endometrial epithelium using both primary hEECs and the endometrial adenocarcinoma cell line Ishikawa. We have observed that, in Ishikawa cells, treatment with butyrate impairs the epithelial barrier, as shown by reduced TEER and increased expression of CLDN2 which, in the gut, was shown to enhance intestinal permeability (Oami *et al.*, 2024). Furthermore, stimulation of both hEECs and Ishikawa cells with a titration of butyrate resulted in increased expression of inflammatory molecules such as CXCL8, IL1β and TNFα, as well as increased expression of antimicrobial peptides of the S100A and β-defensin family. Notably, these effects are observed only when cells are stimulated with either 2 mM or 8 mM butyrate. Acetate stimulation did not significantly increase expression of inflammatory markers or antimicrobial peptides. This might be explained by the presence of higher levels of acetate in the female reproductive tract, as observed in cervicovaginal fluid (Aldunate *et al.*, 2015).

As noted, in women with unexplained infertility undergoing ART who have unsuccessful outcomes, we observed a decrease in *Lactobacillus* spp. abundance and an increase in diversity, with *Prevotella* spp. being significantly increased. In the gut, an increase in *Prevotella* spp. was associated with increased inflammation and butyrate production (Iljazovic *et al.*, 2021). While we could not directly measure microbial-derived metabolites in stored samples, our overall findings lead to the hypothesis that an increase in SCFAs might have occurred and warrants future investigation. Several studies have pointed out that the WOI can be displaced and that measurement of progesterone levels or expression of endometrial markers is still not accurate enough to successfully predict embryo implantation (Enciso *et al.*, 2021). We found that the microbial-derived metabolite butyrate can modify expression of several markers of epithelial receptivity or stromal decidualization. These results, together with reports from other groups (Benner *et al.*, 2018; Hashimoto and Kyono, 2019), suggest that the microbiome plays an important part in shaping endometrial maturation, impacting fertility and implantation rates. We have also shown that increased levels of butyrate can induce inflammation and activate the defence mechanism of endometrial epithelial cells by boosting antimicrobial peptide expression, an important innate immune response to target pathogenic species colonizing the endometrium. Increased expression of inflammatory molecules and antimicrobial peptides suggests that dysbiosis in the endometrium can lead to immune system activation, which, if incorrectly balanced, may prevent successful embryo implantation (Ander *et al.*, 2019). We have previously shown an increase in inflammation as evidenced by aberrant IL-17A pathway activation in women who did not become pregnant (Crosby *et al.*, 2020). Given the role of IL-17A in regulating bacterial colonization (McGeachy *et al.*, 2019), it is possible that the IL-17A pathway could have been activated in these women in an attempt to restore the *Lactobacillus* spp.-dominated microbiome associated with successful implantation.

Limitations of this study included the small sample size for both the cohorts recruited for both the transcriptomic and 16S approach (cohort 1) and the generation of primary hEECs and hESCs (cohort 2). Another limitation of the study was the lack of aneuploidy data for the transferred embryos in cohort 1, however, morphological grading was performed and only embryos of good to top quality were transferred. The lack of embryo ploidy data in our study did not allow us to exclude with certainty embryo ploidy as a possible cause of the failed pregnancy, however with the morphological grading of embryos transferred being good to top quality, we believe that the failure in pregnancy establishment in our cohort is more likely to be attributed to a dysregulation in the endometrial milieu, rather than from an embryonic abnormality. Incorporating ploidy testing in future studies would help clarify this point as preimplantation genetic testing for aneuploidy would identify only euploid embryos that are suitable for the embryo transfer, so this would rule out the embryo quality as the possible cause of pregnancy failure and confirm the dysregulation in the endometrial milieu to be the sole cause of it. The strength of cohort 1, despite being small in terms of sample size, is the inclusion of a nulliparous population meeting very strict criteria and sampling was performed in the secretory stage (day LH + 7), therefore, all the possible confounding variables would be limited thanks to the cohort homogeneity. Notably, the participants recruited for cohort 2 were highly heterogeneous in terms of age and fertility status. The participants in cohort 2 were attending the hospital at different menstrual phases and were undergoing gynaecological investigations for different reasons, such as endometriosis diagnosis (16.7%) or infertility diagnosis (44.4%). To minimize the impact of variability induced by sampling at different menstrual stages on results of the WOI model, the primary cells obtained from the participants in cohort 2 were cultured in the absence of sex hormones, which were only added for the WOI model establishment. It should be noted, however, that the high heterogeneity and the underlying infertility or endometriosis factors of cohort 2 could have had an impact in the transcriptomic results from primary cell stimulations. An increased study cohort size and stratification of samples from women with similar underlying fertility complications in future studies would help delineate this.

Further investigations will be required to accurately define the composition and the metabolic profile of the endometrial microbiome across the menstrual cycle in healthy women. Considering the delicate balance between endometrial receptivity and immune system activation required for successful embryo implantation, it is essential that the impact of the endometrial microbiota on these two physiological activities is established. Once this tight balance is understood, interventions to modify the microbiome in women undergoing ART can be developed.

## Supplementary Material

deaf252_Supplementary_Figure_S1

deaf252_Supplementary_Figure_S2

deaf252_Supplementary_Figure_S3

deaf252_Supplementary_Figure_S4

deaf252_Supplementary_Table_S1

deaf252_Supplementary_Table_S2

deaf252_Supplementary_Table_S3

deaf252_Supplementary_Table_S4

deaf252_Supplementary_Table_S5

deaf252_Supplementary_Table_S6

## Data Availability

The data for the RNA-seq can be found with GEO accession number GSE144895. The data for the 16S-sequencing can be accessed in SRA with the BioProject number PRJNA1338067.
