# Supplementary material for: Contribution of endometrial microbiome to inflammation-mediated infertility in women undergoing ART
Source: Hum Reprod. 2026 Feb 3;41(3):394–409. doi: 10.1093/humrep/deaf252 (PMC13017832; doi:10.1093/humrep/deaf252)
Supplement: deaf252_Supplementary_Figure_S2 [file deaf252_supplementary_figure_s2.pdf]

OXFORD
human reproduction

Human Reproduction, 2026, 1–1
https://doi.org/10.1093/humrep/deaf252
Supplementary Data

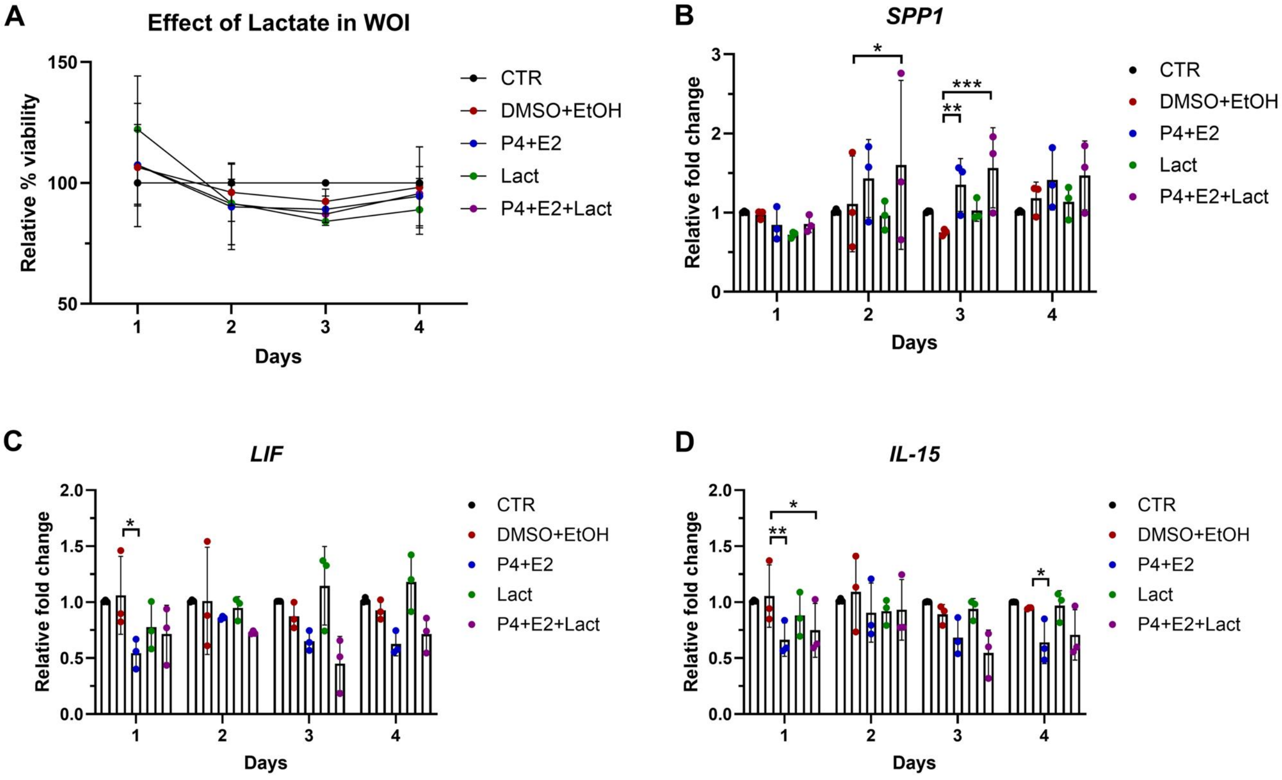

Supplementary Figure S2. Effect of lactate on endometrial receptivity in Ishikawa cells. Ishikawa cells were treated for up to 4 days with P4 (1 µM) and E2 (10 nM) or with equal amounts of dissolving medium controls (DMSO or Ethanol, respectively), with or without 2 mM Lactate. The treatment media was replaced every 2 days. (**A**) Cell viability was assessed by adding to the growing media 10% vol/vol Alamar Blue and reading conversion of resazurin in live cells. Calculation of cell viability was performed using the control cells as reference. (**B–F**) RNA was extracted and expression of SPP1 (**B**), LIF (**C**), IL-15 (**D**) was assessed with qPCR using RPLP0 as reference gene. N = 3. Statistical test applied: Two-way ANOVA with Tukey correction. *$P < 0.05$; **$P < 0.002$; ***$P < 0.0002$. CTR, control; DMSO, dimethyl sulfoxide; E2, oestradiol; EtOH, ethanol; P4, progesterone; Lact, lactate.
