# Supplementary material for: Contribution of endometrial microbiome to inflammation-mediated infertility in women undergoing ART
Source: Hum Reprod. 2026 Feb 3;41(3):394–409. doi: 10.1093/humrep/deaf252 (PMC13017832; doi:10.1093/humrep/deaf252)
Supplement: deaf252_Supplementary_Figure_S3 [file deaf252_supplementary_figure_s3.pdf]

*Human Reproduction*, 2026, 1–1
https://doi.org/10.1093/humrep/deaf252
Supplementary Data

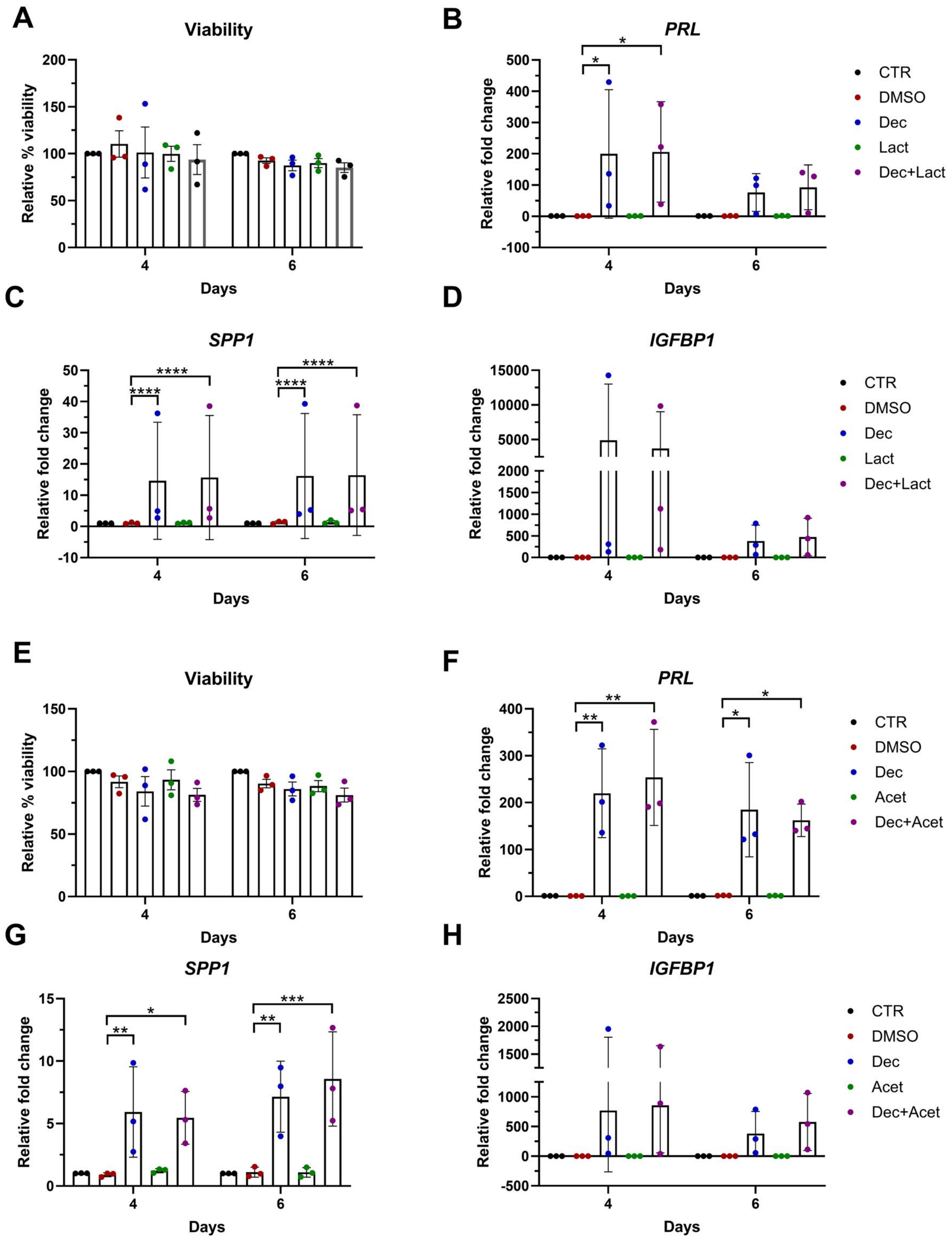


**Supplementary Figure S3. Acetate or Lactate do not impact decidualization marker expression.** Human endometrial stromal cells were isolated from endometrial biopsies. Cells were treated for up to 6 days with decidualization media (P4, 1 μM, and 8-Br-cAMP, 0.1 mg/ml) or with equal amounts of dissolving medium controls (DMSO), in the presence or absence of 2 mM acetate or lactate. Treatment media was refreshed every 2 days. (**A**) and (**E**) Cell viability was assessed by adding 10% vol/vol Alamar Blue and recording resazurin conversion at each timepoint; the relative % viability compared with control cells. RNA was extracted and qPCR was performed using RPLP0 as housekeeping reference gene. Relative gene expression of (**B**) and (**F**) PRL, (**C**) and (**G**) SPP1, (**D**) and (**H**) IGFBP1 N = 3. Statistical test applied: Two-way ANOVA with Dunnet correction. $^*P < 0.05$; $^{**}P < 0.002$; $^{***}P < 0.0002$; $^{****}P < 0.0001$. Acet, acetate; CTR, control; DMSO, dimethyl sulfoxide; EtOH, ethanol; Lact, lactate.
