# Supplementary material for: Contribution of endometrial microbiome to inflammation-mediated infertility in women undergoing ART
Source: Hum Reprod. 2026 Feb 3;41(3):394–409. doi: 10.1093/humrep/deaf252 (PMC13017832; doi:10.1093/humrep/deaf252)
Supplement: deaf252_Supplementary_Figure_S4 [file deaf252_supplementary_figure_s4.pdf]

Human Reproduction, 2026, 1–1
https://doi.org/10.1093/humrep/deaf252
Supplementary Data

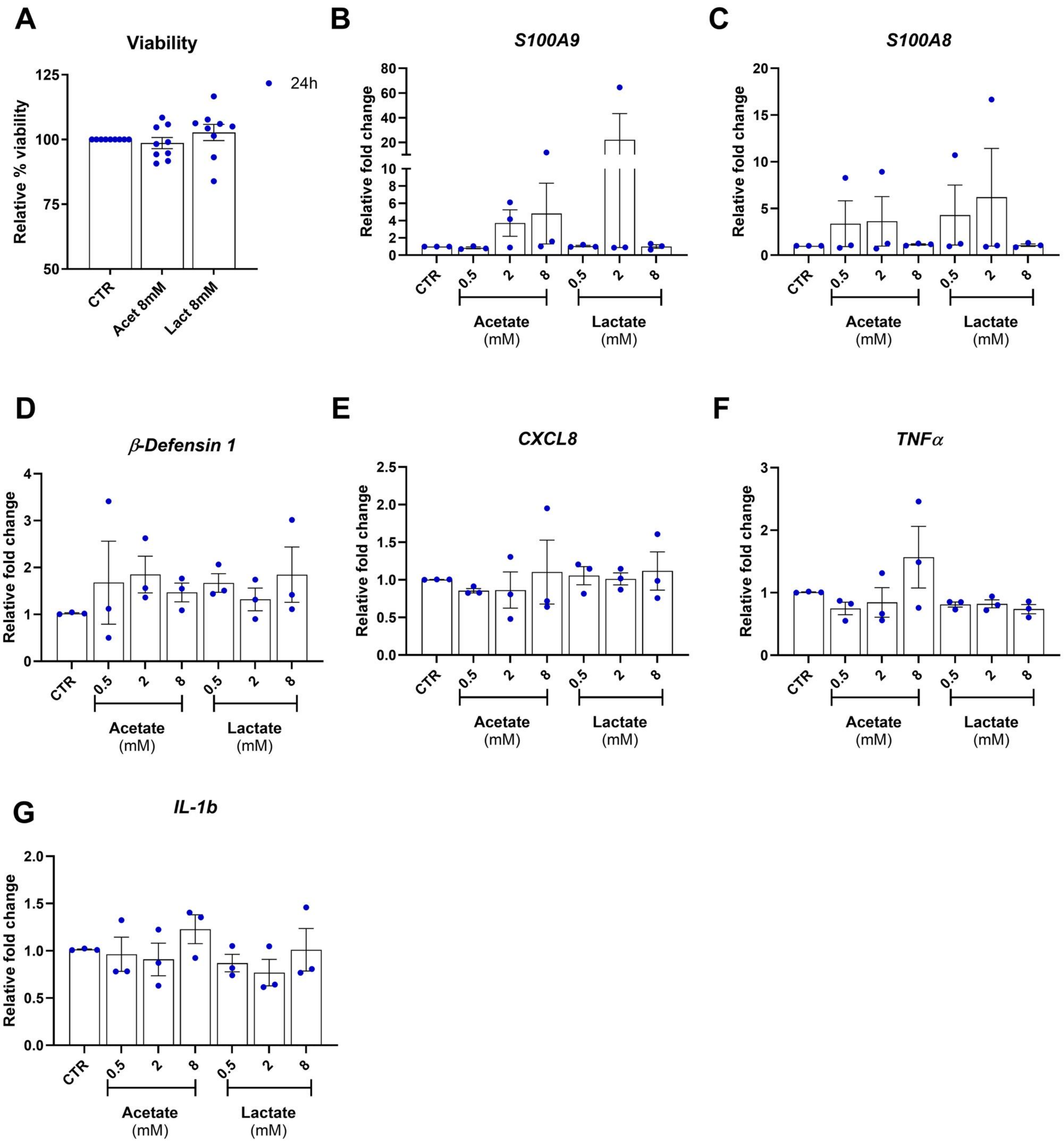

Supplementary Figure S4. Acetate or Lactate do not increase inflammatory markers in hEECs. Human endometrial epithelial cells (hEECs) were isolated from endometrial biopsies. Cells were treated with a titration of acetate or lactate (8, 2, and 0.5 mM) for 24 h. (**A**) Cell viability was assessed by adding 10% vol/vol Alamar blue and reading the optical density of resazurin conversion. Calculation of cell viability was performed using the control cells as reference. (**B–G**) RNA was extracted, and qPCR performed for assessing the changes in expression of antimicrobial peptides ((**B**) S100A9, (**C**) S100A8, and (**D**) human β-defensin 1) and the inflammatory mediators CXCL8, TNFα and IL1β (**E–G**). RPLP0 gene was used as housekeeping reference for qPCR analysis. N ≥ 3. Statistical test applied: Mixed-effects model with Sidak correction. Acet, acetate; CTR, control; Lac, lactate.
