# Supplementary material for: Contribution of endometrial microbiome to inflammation-mediated infertility in women undergoing ART
Source: Hum Reprod. 2026 Feb 3;41(3):394–409. doi: 10.1093/humrep/deaf252 (PMC13017832; doi:10.1093/humrep/deaf252)
Supplement: deaf252_Supplementary_Table_S1 [file deaf252_supplementary_table_s1.pdf]

OXFORD
human reproduction
Human Reproduction, 2026, 1–1
https://doi.org/10.1093/humrep/deaf252
Supplementary Data

Supplementary Table S1. Sequences and concentrations for the primers used in the study.

Target name	Forward primer (5′–3′)	Reverse primer (5′–3′)	Conc (nM)
**RPLP0**	**ACTTGCTGAAAAGGTCAAGGC**	**CCAAATCCCATATCCTCGTCCG**	**700**
IL-23A	CTCAGGGACAACAGTCAGTTC	ACAGGGCTATCAGGGAGCA	700
IL-12B	GCGGAGCTGCTACACTCTC	CCATGACCTCAATGGGCAGAC	700
RORC	GTGGGGACAAGTCGTCTGG	AGTGCTGGCATCGGTTTCG	700
SPP1	GAGGGCTTGGTTGTCAGC	CAATTCTCATGGTAGTGAGTTTTC	700
MUC1	TGCCGCCGAAAGAACTACG	TGGGGTACTCGCTCATAGGAT	100
ITGAV	ATCTGTGAGGTCGAAACAGGA	TGGAGCATACTCAACAGTCTTTG	700
IGFBP1	TTTTACCTGCCAAACTGCAAC	CCCATTCCAAGGGTAGACGC	700
PRL	AAAGGATCGCCATGGAAAG	GCACAGGAGCAGGTTTGAC	700
LIF	CTGTTGGTTCTGCACTGGAA	GCCACATAGCTTGTCCAGGT	500
IL-15	AGAAGCCAACTGGGTGAATG	TACTTGCATCTCCGGACTCA	300
CLDN1	CCAGTCAATGCCAGGTACGAAT	TTGGTGTTGGGTAAGAGGTTGTT	125
CLDN2	CTCCTGGGATTCATTCCTGTT	TCAGGCACCAGTGGTGAGTAG	125
CLDN3	CCACGCGAGAAGAAGTACA	GTAGTCCTTGCGGTCGTAGC	125
TJP1	TGGTGTCCTACCTAATTCAACTCA	CGCCAGCTACAAATATTCCAACA	125
JAMA	CCTGGGAATCTTGGTTTTTG	GGAATGACGAGGTCTGTTTG	125
OCLN	GCTACGGAAGTGGCTATGG	GCGGCAATGAAACAAAAG	125
ECAD	GCCCATTTCCTAAAAACCTG	CTCTGTCACCTTCAGCCATC	125
S100A9	CTCCTCGGCTTTGACAGAGTG	TCTTTTCGCACCAGCTCTTTG	300
S100A8	AGCTGTCTTTCAGAAGACCTG	TCTGCACCCTTTTTCCTGATATAC	300
hBD1	ACCTTCTGCTGTTTACTCTCTGC	TTTGGTAAAGATCGGGCAGGCA	700
CXCL8 (IL-8)	CTCCAAACCTTTCCACCCCA	TCTCAGCCCTCTTCAAAAACTTC	500
TNFα	TGGCCCAGGCAGTCAGATCA	GTAGGAGACGGCGATGCGGC	300
IL1β	GCAGAAGTACCTGAGCTCGC	TGGAAGGAGCACTTCATCTGT	500
MME (CD10)	AGAAGAAACAGCGATGGACTCC	CATAGAGTGCGATCATTGTCACA	700
KRT8	TCCTCAGGCAGCTATATGAAGAG	GGTTGGCAATATCCTCGTACTGT	300
MCT1	GGGTTATAAGGCAGCCTCGCT	TCCAACTGCTGGTGGCATTTT	500
MCT4	GAGTTTGGGATCGGCTACAG	CGGTTCACGCACACACTG	500
