# Supplementary material for: Contribution of endometrial microbiome to inflammation-mediated infertility in women undergoing ART
Source: Hum Reprod. 2026 Feb 3;41(3):394–409. doi: 10.1093/humrep/deaf252 (PMC13017832; doi:10.1093/humrep/deaf252)
Supplement: deaf252_Supplementary_Table_S2 [file deaf252_supplementary_table_s2.pdf]

OXFORD

human reproduction

Human Reproduction, 2026, 1–1
https://doi.org/10.1093/humrep/deaf252
Supplementary Data

Supplementary Table S2. Linear regression analysis of the diversity indexes retrieved from the 16S sequencing and the counts per million expression values for the receptivity markers from the RNA-seq (Crosby *et al.*, 2020).

Linear regression of 16S-seq-derived beta diversity indexes and counts per million from the RNA-seq

	SPP1	PRL	IGFBP1	MUC1	LIF	IL15	ITGAV
**Goodness of fit**							
**R square**	0.075	0.04086	0.08837	0.0103	0.0002333	0.004873	0.2219
**Sy.x**	6958	47.13	3737	3332	806.9	514.7	661.4
**Is slope significantly non-zero?**							
**F**	1.297	0.6815	1.551	0.1665	0.003734	0.07836	4.563
**DFn, DFd**	1, 16	1, 16	1, 16	1, 16	1, 16	1, 16	1, 16
**P-value**	0.2715	0.4212	0.2309	0.6886	0.9520	0.7831	0.0485
**Deviation from zero?**	ns	ns	ns	ns	ns	ns	*

Linear regression of 16S-seq-derived Shannon diversity indexes and counts per million from the RNA-seq

	SPP1	PRL	IGFBP1	MUC1	LIF	IL15	ITGAV
**Goodness of fit**							
**R square**	0.06074	0.00001211	0.004833	0.02099	0.0008808	0.06874	0.01505
**Sy.x**	9089	48.76	3898	3570	920.2	561.6	738.2
**Is slope significantly non-zero?**							
**F**	1.035	0.0001937	0.07771	0.343	0.01411	1.181	0.2445
**DFn, DFd**	1, 16	1, 16	1, 16	1, 16	1, 16	1, 16	1, 16
**P-value**	0.3242	0.9891	0.784	0.5663	0.9069	0.2933	0.6277
**Deviation from zero?**	ns	ns	ns	ns	ns	ns	ns

Linear regression of 16S-seq-derived Simpson diversity indexes and counts per million from the RNA-seq

	SPP1	PRL	IGFBP1	MUC1	LIF	IL15	ITGAV
**Goodness of fit**							
**R square**	0.05532	0.008026	0.01243	0.02165	0.00006458	0.02339	0.03504
**Sy.x**	9115	48.57	3884	3568	920.6	575.1	730.7
**Is slope significantly non-zero?**							
**F**	0.9369	0.1295	0.2014	0.354	0.001033	0.3831	0.5811
**DFn, DFd**	1, 16	1, 16	1, 16	1, 16	1, 16	1, 16	1, 16
**P-value**	0.3475	0.7237	0.6596	0.5602	0.9748	0.5447	0.457
**Deviation from zero?**	ns	ns	ns	ns	ns	ns	ns

The correlations graphed in Figure 4A, B and C (upper panel) are highlighted in green.
