# Supplementary material for: Contribution of endometrial microbiome to inflammation-mediated infertility in women undergoing ART
Source: Hum Reprod. 2026 Feb 3;41(3):394–409. doi: 10.1093/humrep/deaf252 (PMC13017832; doi:10.1093/humrep/deaf252)
Supplement: deaf252_Supplementary_Table_S3 [file deaf252_supplementary_table_s3.pdf]

OXFORD
human reproduction

Human Reproduction, 2026, 1–1
https://doi.org/10.1093/humrep/deaf252
Supplementary Data

Supplementary Table S3. Linear regression analysis for receptivity markers (RNA-seq) showing significant correlation with microbiota diversity indexes retrieved from 16S sequencing, analysed by pregnancy status (either Pregnant or Not Pregnant) (Crosby *et al.*, 2020).

Linear regression of 16S-seq-derived beta diversity indexes and counts per million from the RNA-seq

	ITGAV—Pregnant	ITGAV—Not pregnant
**Goodness of fit**		
**R square**	0.2219	0.3309
**Sy.x**	661.4	646.8
**Is slope significantly non-zero?**		
**F**	4.563	3.461
**DFn, DFd**	1, 16	1, 7
**P-value**	0.0485	0.1052
**Deviation from zero?**	*	ns

Linear regression of 16S-seq-derived Shannon diversity indexes and counts per million from the RNA-seq

	SPP1—Pregnant	SPP1—Not pregnant
**Goodness of fit**		
**R square**	0.5859	0.04577
**Sy.x**	5788	9191
**Is slope significantly non-zero?**		
**F**	8.490	0.3837
**DFn, DFd**	1, 6	1, 8
**P-value**	0.0269	0.5528
**Deviation from zero?**	*	ns

Linear regression of 16S-seq-derived Simpson diversity indexes and counts per million from the RNA-seq

	SPP1—Pregnant	SPP1—Not pregnant
**Goodness of fit**		
**R square**	0.5235	0.06597
**Sy.x**	6209	9094
**Is slope significantly non-zero?**		
**F**	6.591	0.5650
**DFn, DFd**	1, 6	1, 8
**P-value**	0.0425	0.4738
**Deviation from zero?**	*	ns

Results from these correlations are graphed in Fig. 4A, B, and C (bottom panel) and are highlighted in light red for 'pregnant' and blue for 'not pregnant'.
