# Supplementary material for: Contribution of endometrial microbiome to inflammation-mediated infertility in women undergoing ART
Source: Hum Reprod. 2026 Feb 3;41(3):394–409. doi: 10.1093/humrep/deaf252 (PMC13017832; doi:10.1093/humrep/deaf252)
Supplement: deaf252_Supplementary_Table_S4 [file deaf252_supplementary_table_s4.pdf]

Human Reproduction, 2026, 1–1
https://doi.org/10.1093/humrep/deaf252
Supplementary Data

Supplementary Table S4. Linear regression analysis examining the relationship between percentage abundance of *Prevotella* spp. and Lactobacillus *spp*. retrieved from 16S sequencing and receptivity marker expression (counts per million) from endometrial bulk RNA-seq analysis (Crosby *et al.*, 2020).

Linear regression of 16S-seq-derived *Prevotella* spp. percentage and counts per million from the RNA-seq

	SPP1	PRL	IGFBP1	MUC1	LIF	IL15	ITGAV
**Goodness of fit**							
**R square**	0.0534	0.003773	0.00577	0.0000030	0.04697	0.04102	0.1307
**Sy.x**	8726	46.22	3685	3442	865.1	552	668.9
**Is slope significantly non-zero?**							
**F**	1.015	0.06818	0.1046	5.457e-005	0.8872	0.7699	2.706
**DFn, DFd**	1, 18	1, 18	1, 18	1, 18	1, 18	1, 18	1, 18
**P-value**	0.3269	0.7970	0.7501	0.9942	0.3587	0.3918	0.1173
**Deviation from zero?**	ns	ns	ns	ns	ns	ns	ns

Linear regression of 16S-seq-derived *Lactobacillus* spp. percentage and counts per million from the RNA-seq

	SPP1	PRL	IGFBP1	MUC1	LIF	IL15	ITGAV
**Goodness of fit**							
**R square**	0.003778	0.02802	0.02474	0.003749	0.000611	0.01276	0.1101
**Sy.x**	8952	45.65	3650	3436	885.9	560.1	676.7
**Is slope significantly non-zero?**							
**F**	0.06827	0.5188	0.4567	0.06773	0.01101	0.2326	2.227
**DFn, DFd**	1, 18	1, 18	1, 18	1, 18	1, 18	1, 18	1, 18
**P-value**	0.7968	0.4806	0.5078	0.7976	0.9176	0.6354	0.1529
**Deviation from zero?**	ns	ns	ns	ns	ns	ns	ns
