# Supplementary material for: Contribution of endometrial microbiome to inflammation-mediated infertility in women undergoing ART
Source: Hum Reprod. 2026 Feb 3;41(3):394–409. doi: 10.1093/humrep/deaf252 (PMC13017832; doi:10.1093/humrep/deaf252)
Supplement: deaf252_Supplementary_Table_S5 [file deaf252_supplementary_table_s5.pdf]

OXFORD
human reproduction
Human Reproduction, 2026, 1–1
https://doi.org/10.1093/humrep/deaf252
Supplementary Data

Supplementary Table S5. Linear regression analysis of the diversity indexes retrieved from 16S sequencing and the counts per million expression values from bulk RNA-seq (Crosby *et al.*, 2020).

Linear regression of 16S-seq-derived Shannon diversity indexes and counts per million from the RNA-seq

	S100A9	S100A8	DEFB1	PI3	CXCL8	TNFa	IL-1b	IL-1a
**Goodness of fit**								
**R square**	0.01417	0.05137	0.012	0.1849	0.07007	0.06714	0.02275	0.01889
**Sy.x**	105.8	15.1	7059	4.663	40.03	2.125	40.23	11.97
**Is slope significantly non-zero?**								
**F**	0.2299	0.8664	0.1944	3.63	1.206	1.152	0.3725	0.308
**DFn, DFd**	1, 16	1, 16	1, 16	1, 16	1, 16	1, 16	1, 16	1, 16
**P-value**	0.6381	0.3658	0.6652	0.0749	0.2885	0.2991	0.5502	0.5866
**Deviation from zero?**	ns	ns	ns	ns	ns	ns	ns	ns

Linear regression of 16S-seq-derived Simpson diversity indexes and counts per million from the RNA-seq

	S100A9	S100A8	DEFB1	PI3	CXCL8	TNFa	IL-1b	IL-1a
**Goodness of fit**								
**R square**	0.008215	0.05393	0.01469	0.1314	0.08186	0.03547	0.02403	0.009054
**Sy.x**	106	15.08	7049	4.813	39.77	2.161	40.2	12.03
**Is slope significantly non-zero?**								
**F**	0.1574	0.912	0.2386	2.421	1.427	0.5884	0.394	0.1462
**DFn, DFd**	1, 19	1, 16	1, 16	1, 16	1, 16	1, 16	1, 16	1, 16
**P-value**	0.696	0.3538	0.6319	0.1393	0.2497	0.4542	0.5391	0.7072
**Deviation from zero?**	ns	ns	ns	ns	ns	ns	ns	ns

Linear regression of 16S-seq-derived beta-diversity indexes and counts per million from the RNA-seq

	S100A9	S100A8	DEFB1	PI3	CXCL8	TNFa	IL-1b	IL-1a
**Goodness of fit**								
**R square**	0.02333	0.1129	0.04888	0.02212	0.06611	2.55E-05	0.01612	0.02436
**Sy.x**	71.44	14.96	6835	4.7	40.01	2.589	40.55	11.88
**Is slope significantly non-zero?**								
**F**	0.3822	2.037	0.8222	0.362	1.133	0.000408	0.2622	0.3994
**DFn, DFd**	1, 16	1, 16	1, 16	1, 16	1, 16	1, 16	1, 16	1, 16
**P-value**	0.5452	0.1727	0.378	0.5559	0.303	0.9841	0.6156	0.5363
**Deviation from zero?**	ns	ns	ns	ns	ns	ns	ns	ns
