# Supplementary material for: Contribution of endometrial microbiome to inflammation-mediated infertility in women undergoing ART
Source: Hum Reprod. 2026 Feb 3;41(3):394–409. doi: 10.1093/humrep/deaf252 (PMC13017832; doi:10.1093/humrep/deaf252)
Supplement: deaf252_Supplementary_Table_S6 [file deaf252_supplementary_table_s6.pdf]

Human Reproduction, 2026, 1–1
https://doi.org/10.1093/humrep/deaf252
Supplementary Data

Supplementary Table S6. Linear regression analysis of the percentage abundance of *Prevotella* spp. and *Lactobacillus* spp. retrieved from the 16S sequencing and the counts per million expression values from bulk RNA-seq (Crosby *et al.*, 2020).

Linear regression of 16S-seq-derived *Prevotella* spp. percentage and counts per million from the RNA-seq

	S100A9	S100A8	DEFB1	PI3	CXCL8	TNFa	IL-1b	IL-1a
**Goodness of fit**								
**R square**	0.003639	0.001561	0.07051	0.008802	0.01299	0.09233	0.004987	0.04953
**Sy.x**	102.1	15.06	6677	4.984	39.16	2.419	38.48	11.26
**Is slope significantly non-zero?**								
**F**	0.06573	0.02813	1.365	0.1598	0.2369	1.831	0.09022	0.9381
**DFn, DFd**	1, 18	1, 18	1, 18	1, 18	1, 18	1, 18	1, 18	1, 18
**P-value**	0.8006	0.8687	0.2579	0.694	0.6323	0.1928	0.7673	0.3456
**Deviation from zero?**	ns	ns	ns	ns	ns	ns	ns	ns

Linear regression of 16S-seq-derived *Lactobacillus* spp. percentage and counts per million from the RNA-seq

	S100A9	S100A8	DEFB1	PI3	CXCL8	TNFa	IL-1b	IL-1a
**Goodness of fit**								
**R square**	0.07513	0.103	0.01347	0.1003	0.04076	0.002305	0.03806	0.01309
**Sy.x**	98.34	14.28	6879	4.749	38.61	2.536	37.83	11.47
**Is slope significantly non-zero?**								
**F**	1.462	2.067	0.2457	2.007	0.765	0.04158	0.7121	0.2388
**DFn, DFd**	1, 18	1, 18	1, 18	1, 18	1, 18	1, 18	1, 18	1, 18
**P-value**	0.2422	0.1677	0.6261	0.1737	0.3933	0.8407	0.4098	0.631
**Deviation from zero?**	ns	ns	ns	ns	ns	ns	ns	ns

Crosby, D.A., L.E. Glover, E.P. Brennan, P. Kelly, P. Cormican, B. Moran, F. Giangrazi, P. Downey, E.E. Mooney, B.J. Loftus, F.M. McAuliffe, M. Wingfield, C. O'Farrelly and D.J. Brennan. Dysregulation of the interleukin-17A pathway in endometrial tissue from women with unexplained infertility affects pregnancy outcome following assisted reproductive treatment. Hum Reprod (2020).

© The Author(s) 2026. Published by Oxford University Press on behalf of European Society of Human Reproduction and Embryology.
This is an Open Access article distributed under the terms of the Creative Commons Attribution License (https://creativecommons.org/licenses/by/4.0/), which permits unrestricted reuse, distribution, and reproduction in any medium, provided the original work is properly cited.
Human Reproduction, 2026, 00, 1–16
https://doi.org/10.1093/humrep/deaf252
Original Article
